# Diversity and Applications of Endophytic Actinobacteria of Plants in Special and Other Ecological Niches

**DOI:** 10.3389/fmicb.2018.01767

**Published:** 2018-08-08

**Authors:** Radha Singh, Ashok K. Dubey

**Affiliations:** Division of Biological Sciences and Engineering, Netaji Subhas Institute of Technology, New Delhi, India

**Keywords:** endophytic actinobacterial divsersity, special habitats, chemical diversity, secondary metabolites, therapeutics, plant growth promotion, phytopathogens

## Abstract

Actinobacteria are wide spread in nature and represent the largest taxonomic group within the domain Bacteria. They are abundant in soil and have been extensively explored for their therapeutic applications. This versatile group of bacteria has adapted to diverse ecological habitats, which has drawn considerable attention of the scientific community in recent times as it has opened up new possibilities for novel metabolites that may help in solving some of the most challenging problems of the day, for example, novel drugs for drug-resistant human pathogens, affordable means to maintain ecological balance in various habitats, and alternative practices for sustainable agriculture. Traditionally, free dwelling soil actinobacteria have been the subject of intensive research. Of late, symbiotic actinobacteria residing as endophytes within the plant tissues have generated immense interest as potential source of novel compounds, which may find applications in medicine, agriculture, and environment. In the light of these possibilities, this review focuses on the diversity of endophytic actinobacteria isolated from the plants of extreme habitats and specific ecological niches. Furthermore, an attempt has been made to assign chemical class to the compounds obtained from endophytic actinobacteria. Potential therapeutic applications of these compounds and the utility of endophytic actinobacteria in agriculture and environment are discussed.

## Introduction

Actinobacteria are Gram-positive bacteria with high G+C content, which are filamentous with substrate and aerial mycelia. They rose to prominence during the hunt for drugs from microbes following the discovery of Penicillin from *Penicillium notatum* by Alexander Fleming. They are now well established as prolific producers of a wide range of bioactive secondary metabolites such as antibiotics, enzymes, enzyme inhibitors, antioxidants, and others having therapeutic significance (Barka et al., 2016). Approximately, 22,000 bioactive secondary metabolites of microbial origin have been reported, of which fifty percent are from actinobacteria only. Approximately 160 antibiotics are being currently used in human therapy and in agriculture (Berdy, 2012). However, this is a small number to meet the ever-growing requirement for such compounds. Therefore, there is an urgent need for further exploration of actinobacteria to expand the repertoire of bioactive molecules. Since only a small part of the bioactive molecules isolated from actinobacteria and their biosynthetic gene clusters are studied, bioprospecting of actinobacteria for bioactive molecules holds a great promise.

In view of the emerging threat to deal with drug resistant pathogens, there is an increasing sense of urgency for discovery and development of new drugs. In order to address this challenge, novel approaches need to be devised for searching new molecules. One of which is “renaissance in antibacterial discovery from actinomycetes” (Baltz, 2008). Search of special ecological niches along with new methods of isolation of novel genera/species of actinobacteria may lead to the identification of new gene clusters, and hence, new products (Xu et al., 2010). Researchers are now focusing on the isolation of actinobacteria from diverse habitats like oceans (Subramani and Aalbersberg, 2013), extreme environments (Tang et al., 2003; Hamedi et al., 2013), inner tissues of plants (Chankhamhaengdecha et al., 2013), animals excreta (Cao et al., 2012), algae, and lichens (González et al., 2005; Yamamura et al., 2011). Hence, it is highly encouraging to explore the actinobacteria inhabiting special niches like extremophilic plants for discovering hitherto unexploited strains.^1^,^2^,^3^

For centuries plants have been extensively used as the sources of bioactive compounds for therapeutic purposes. In the recent times, plant associated microorganisms have been shown to produce compounds of high therapeutic value (Subbulakshmi et al., 2012). The microorganisms residing inside plant tissues, mostly in symbiotic relationship, may include different groups, for example, fungi and bacteria including actinobacteria (Pimentel et al., 2011; Singh and Dubey, 2015). The endophytes complete their life cycle within the host plants, normally without subjecting them to any disadvantage. Endophytes are ubiquitous in nature and remain in specific association with the host plants, for example, mutualism or antagonism but not parasitism (Nair and Padmavathy, 2014). They play significant roles in enhancing growth of the host plants by producing phytohormones and other growth promoting factors. In turn, they are benefited with nutrients and shelter within the host plant. They also improve the host's tolerance for abiotic and biotic stresses, while offering resistance against insects, pests, and pathogens.

Studies revealed that nearly all the plants harbor endophytes (Strobel and Daisy, 2003). These microbes attain a very special niche in plants by colonizing themselves in stem, root, petioles, leaf segments, inflorescence of weeds, fruit, buds, seeds along with dead, and hollow hyaline cells (Hata and Sone, 2008; Stepniewska and Kuzniar, 2013). However, the colonization of endophytes in different plants is highly variable depending on factors like host specificity, developmental stage of the host, geographical conditions, and the extant microbial diversity (Dudeja and Giri, 2014). Extensive and intensive studies are, however, required to interpret the relationship between the host and the endophytes, and effect of such interactions on the production of bioactive metabolites.

Considering the immense diversity of the flora and widespread plant-microbe associations, the structural and functional diversity among endophytic actinobacteria is expected to be immense. Such diverse group of endophytic actinobacteria could be source of novel biomolecules with myriad of applications. This review highlights the occurrence and diversity of endophytic actinobacteria in the plants from special ecological niches: arid zones, mangrove, and saline ecosystems, and aquatic habitats. Furthermore, chemical and structural diversity of the metabolites reported from endophytic actinobacteria and their potential applications in medicine, agriculture and environment are discussed.

## Origin and emerging concepts of endophytes

Majority of the microbes colonizing the plants internally play key roles in plant's fitness and growth. A minor fraction of them may also cause diseases (Andreote et al., 2014). The overall interaction, however, is mutually beneficial (Mendes et al., 2013; Philippot et al., 2013). Thousands of microorganisms can be the inhabitants of a single plant either in the form of epiphytes in the phyllospheric region or as endophytes within tissues of leaves, roots, or stems. Endophytes display extensive diversity (Turner et al., 2013; Andreote et al., 2014).

The presence of microbial cells in the plant tissue was first observed by De Bary (1866), who coined the term endophytes. The study and exploration of these microbes remained ignored for long. De Bary defined endophytes as “any organism that grows within plant tissue.” Subsequently, the definition had been modified as the studies on endophytes progressed. The most appropriate and comprehensive of the definitions states that endophytes include a suite of microorganisms that grow intra or inter-cellularly in the tissues of the plants without causing any harmful effect to them.

The endophytic communities have been categorized into different subgroups, such as “obligate” or “facultative” (Rosenblueth and Martínez-Romero, 2006). Obligate endophytes are the microbes that depend solely on the metabolism of plants for survival, and whose transmission amongst plants take place by the action of various vectors or by vertical transmission (Hardoim et al., 2008). On the other hand, the facultative endophytes spend certain stages of their life cycle independent of the host plant. They are indirectly associated with plants through neighboring soil environment and atmosphere (Abreu-Tarazi et al., 2010). As the studies advanced, more hypotheses appeared about the origin of endophytic organisms. Extensive studies were undertaken to find out the origin of endophytic organisms in different species of plants (Hallmann et al., 1997; Mitter et al., 2013). Microorganisms associated with the rhizosphere and with the seeds were considered to be the major source of endophytes. Studies based on genome organization revealed the specific endophytes-plant interactions in terms of plant specificity, abundance, and mode of transmission (Andreote et al., 2014). However, in order to understand the relationship of endophytes and the host-plants, scientists have thoroughly studied the genome sizes and origins of endophytes and compared with those of bacteria and their lifestyles (Dini-Andreote et al., 2012). Studies revealed that the genome size of endophytic microbes were smaller than those of free leaving microbes. Presence of less number of mobile genetic elements accounted for the smaller size but increased genomes stability. These observations suggested that microbes with smaller and stable genome are more likely to establish endophytic association (Mitter et al., 2013). It has been observed that the association between the endophyte and its host-plant begins at very early stage of the plant development (Hasegawa et al., 2006). With advancement in the research on plant-microbe interactions, the definitions were modified to offer a clearer and comprehensive description of the endophytes.

## Endophytic actinobacterial diversity in various ecosystems

Actinobacteria are predominantly free-living microorganisms found in diverse environments. Soil is the most dominant reservoir for actinobacteria and also represnts the zone of most active interaction between the actinobacteria and the root system of plants. On account of such interactions, the roots of plants can be considered to be the richest source of the endophytic actinobacteria. Studies have demonstrated that plant roots influence the soil region in their vicinity through exudates, which significantly impact plant-microbe interactions in the rhizosphere (Schenk et al., 2012).

Diversity of endophytic actinobacteria had been reviewed by several groups in recent times. Dinesh et al. (2017) had discussed endophytic actinobacteria from terrestrial plants, which included mainly medicinal plants. Nalini and Prakash (2017), Golinska et al. (2015) and Masand et al. (2015) had also reviewed the diversity of endophytic actinobacteria in medicinal plants. Qin et al. (2011) discussed biodiversity of endophytic actinobacteria from crop plants, medicinal plants, Chinese cabbage, and plants from tropical rain forests of China. It had been reported that medicinal plants of tropical rain forests were the richest source of novel endophytic actinobacteria (Qin et al., 2009). Evidently, endophytic actinobacteria from medicinal plants and tropical rain forests have drawn considerable attention of the scientific community till date. But such actinobacteria from the plants of some of the fascinating ecological niches, for example, arid zones, high salt zones (mangroves and halophytes), and aquatic ecosystems have not yet been specifically reviewed. An attempt has, therefore, been made to address this gap in the present review, wherein endophytic actinobacteria from the plants of special niches have been considered. The plants growing in the environments that are under abiotic stress have evolved to cope up with such factors like temperature, humidity, drought, high rainfall, soil salinity, nutrition limitations, and others. These plants are evolutionarily adapted to thrive in the environments characterized by a high degree of abiotic stresses. Physiological features like modification in the root and shoot systems, structure of leaves and anatomical changes like structure of cortex, xylem and phloem aid such plants to survive in conditions like drought, water logging, marshy and salinity. These plants are also referred to as extremophiles due to their ability to thrive in these extreme environmental conditions. Altered environmental conditions are likely to induce changes in plant physiology. Also, there is difference in the chemo-attractants or signal compounds as well as a change in nutrient availability (Kandeler et al., 2006; Haase et al., 2007). These alterations cause changes in the activity and the diversity of microbial communities associated with the plants (Drigo et al., 2009).

Ubiquitous occurrence of the endophytic actinobacteria is evident from their discovery from the plants of diverse climates: arid zones, saline habitats, aquatic ecosystems, and other ecological niches. They have been reported to colonize any tissue or organ of the host plant (Dinesh et al., 2017). It has been noted that different tissues and organs of the plant are colonized by different actinobacteria, which might be determined by the host-microbe interaction. Such processes, which seem to confer a level of selectivity, may impact the endophytic actinobacterial diversity (Nimnoi et al., 2010). From the reports available till date, it is evident that endophytic actinobacteria are abundant in roots, occur moderately in the stems, and have been found in least numbers in the leaves (Gangwar et al., 2014). Such a distribution pattern for the endophytic actinobacteria seems rational since the roots have maximum exposure to and interactions with the microbial population in the rhizosphere. The endophytic actinobacteria are broadly considered as *Streptomyces* spp. and non-*Streptomyces* spp. The non-*Streptomyces* spp. are uncommon and are classified as rare taxa.

Similarly, the variation in climatic conditions contributes to greater diversity in their flora and the resident microbial communities. For example, the microbial communities of tropical and temperate regions possess more diverse range of endophytes (Strobel and Daisy, 2003). Studies have revealed that physiological diversity is linked to the geographical diversity of the host plants (Du et al., 2013). Despite the relationship between abundance and diversity of endophytic actinobacteria and plants, no direct correlation among the host plants and their endophytic communities could be established. *Actinosynnema*, isolated from a grass blade, was the first probable actinobacterium of plant origin (Hasegawa et al., 1978). Afterwards, there have been numerous reports on the isolation of endophytic actinobacteria from various plant sources as discussed in the ensuing sections.

### Diversity of endophytic actinobacteria in arid ecosystems

The flora of arid ecosystem remains under continuous abiotic stress like draft and salt accumulation, which induces the development of physiological and molecular stress responses in them. This allows the plants to grow normally under such harsh environment. Aridification is a worldwide problem in agriculture and crop production. It has been found that there is a huge built-up of microorganisms that support the plants to cope up with such environments by developing adaptation strategies (Asaf et al., 2017). It is evident from literature that plant-associated extremophilic and extremotolerant actinobacteria comprised the group of less investigated microbes. It has been reported that actinobacteria residing in deserts display immense capability to survive under adverse conditions of pH or salinity and possess noteworthy gene clusters to produce bioactive compounds (Mohammadipanah and Wink, 2016). A very few reports are, however, available on the endophytic actinobacteria from arid plants, thus there are good opportunities to study and explore diversity of new microbial species in the plants from arid zones (Thumar et al., 2010).

Huang et al. (2012) reported actinobacteria from arid plants, which included several genera like *Streptomyces, Micromonospora, Nocardia, Nonomuraea*, and *Amycolatopsis*. Drought tolerant endophytic actinobacteria, *Streptomyces coelicolor* DE07, *Streptomyces olivaceus* DE10 and *Streptomyces geysiriensis* DE27 are some of the endophytic actinobacteria reported in the plants of arid regions (Yandigeri et al., 2012). Goudjal et al. (2014) reported 22 *Streptomyces* spp. and five non-*Streptomyces* spp. on the basis of morphology and chemotaxonomic analyses, isolated randomly from five plants well adapted to the poor sandy soil and arid climatic conditions of the Algerian Sahara. *Streptomyces mutabilis* strain IA1, isolated from Saharan soil, was another endophyte as it colonized inside the caryopsis up to the endocarp layer in the wheat plant (Toumatia et al., 2016). Wang et al. (2015a,b) had reported isolation of novel endophytic actinobacteria like *Frigoribacterium endophyticum* and *Labedella endophyticum* from the roots of plant *Anabis eliator* from Urumqi (cold arid region), China. Novel endophyte, *Streptomyces zhaozhouensis* was isolated from *Candelabra aloe* which is a succulent collected from Zhaozhou, China (He et al., 2014). A novel species, *Streptomyces ginkgonis* has been isolated from the seeds of *Ginkgo biloba* (Yan et al., 2017). *Glycomyces anabasis*, a novel endophytic actinobacterium had been reported from the roots of *Anabasis aphylla* L of arid region in China (Zhang et al., 2018). In a study on endophytic actinobacteria from the plant *Ferula sinkiangensis* K. M. Shen, 125 endophytic strains were isolated from the roots. These actinobacteria belonged to 3 phyla, 13 orders, 23 families and 29 genera which contained potential novel species (Liu Y. et al., 2017). Plant growth promoting actinobacteria belonging to genus *Amycolatopsis* were also identified during this work. Metagenomic study of arid soil has shown the presence of the genera *Streptomyces, Micromonospora, Saccharothrix, Streptosporangium, Cellulomonas, Amycolatopsis, Geodermatophilus, Lechevalieria, Nocardia*, and *Actinomadura*, but no such reports are available on endophytes from the arid plants (Mohammadipanah and Wink, 2016). *Streptomyces, Actinoallomurus, Amycolatopsis, Kribbella*, and *Microbispora* were isolated from healthy roots of wattle tree, *Acacia auriculiformis* (Thamchaipenet et al., 2010). Despite water and nutrient deprived conditions, the flora of arid region was inhabited by many endophytic actinobacteria that included majorly *Streptomyces*, followed by other rare genera and novel species (Table 1).

**Table 1:** Endophytic actinobacterial diversity in plants of special habitats.

**Habitat**	**Host plant**	**Endophytic actinobacteria**	**References**
**CROPS PLANTS**			
Saline	*Limonium sinense*	*Kineococcus endophytica* KLBMP 1274^T^, *Streptomyces* sp. KLBMP 5084, *Glutamicibacter halophytocola* sp. nov. KLBMP 5180	Bian et al., 2012a; Feng et al., 2017
Arid	*Lupinus termis*	*Actinoplane missouriensis* CPWT, CPNWT, CNPM	El-Tarabily, 2003
Arid	*Triticum aestivum*	*Streptomyces* sp.*, Microbispora* sp.*, Micromonospora* sp.*, Nocardioides* sp.	Coombs and Franco, 2003
Arid	*Curcuma phaeocaulis*	*Streptomyces phytohabitans* KLBMP 4601^T^	Bian et al., 2012b
Arid (Cold)	*Solanum melongena*	*Nonomuraea solani* NEAU-Z6^T^	Wang et al., 2013
Arid	*Glycine max*	*Actinoplanes hulinensis* NEAU-M9^T^, *Streptomyces harbinensi* NEAU-Da3^T^*, Wangella harbinensis* NEAU-J3^T^	Jia et al., 2013; Liu et al., 2013; Shen et al., 2013
Aquatic	*Oryza sativa* (Thai jasmine rice plant)	*Actinoallomurus oryzae* sp. nov. GMKU 370^T^	Indananda et al., 2011
**WOODY PLANTS**			
Arid	*Acacia auriculiformis*	*Actinoallomurus acaciae* GMKU 931^T^, *Streptomyces* sp. GMKU 937, GMKU 940, *Actinoallomurus coprocola* GMKU 943, *Amycolatopsis tolypomycina* GMKU 932, *Kribbella* sp. GMKU 938, *Microbispora mesophila* GMKU 941 and GMKU 942	Bunyoo et al., 2009; Thamchaipenet et al., 2010
Arid	*Eucalyptus microcarpa*	*Promicromonospora endophytica* EUM 273^T^	Kaewkla and Franco, 2013
Arid	*Camptotheca acuminate*	*Blastococcus endophyticus* YIM 68236^T^, *Plantactinospora endophytica* YIM 68255^T^	Zhu et al., 2012, 2013
Semi- Arid	*Dracaena cochinchinensis*	*Streptomyces* sp. (HUST 001, HUST 011, HUST 014, 015, 018) *Nocardiopsis* sp. HUST 017, *Pseudonocardia* sp. HUST 013	Salam et al., 2017
Mangrove (Lowlands)	*Aquilaria crassna*	*Streptomyces javensis* GQ179657*, Nonomuraea rubra* GQ179656*, Actinomadura glauciflava* GQ179654*, Pseudonocardia halophobica* GQ179660*, Nocardia alba* GQ179653	Nimnoi et al., 2010
Mangrove	*Xylocarpus granatum*	*Jishengella endophytica* 202201^T^	Xie et al., 2011
Mangrove	*Avicennia marina, Aegiceras corniculatum, Kandelia obovota, Bruguiera gymnorrhiza, and Thespesia populnea*	*Streptomyces* sp., *Curtobacterium* sp., *Mycobacterium* sp., *Micrococcus* sp*., Brevibacterium* sp., *Kocuria* sp., *Nocardioides* sp*., Kineococcus* sp., *Kytococcus* sp., *Marmoricola* sp., *Microbacterium* sp*. Micromonospora*, sp., *Actinoplanes* sp*., Agrococcus* sp.*, Amnibacterium* sp., *Brachybacterium* sp., *Citricoccus* sp., *Dermacoccus* sp., *Glutamicibacter* sp., *Gordonia* sp., *Isoptericola* sp., *Janibacter* sp., *Leucobacter* sp., *Nocardia* sp., *Nocardiopsis* sp., *Pseudokineococcus*, sp., *Sanguibacter* sp., *Verrucosispora* sp.	Jiang et al., 2018
Mangrove	*Thespesia populnea*	*Marmoricola endophyticus* 8BXZ-J1^T^	Jiang et al., 2017
**MEDICINAL PLANTS**			
Arid	*Lobelia clavatum*	*Pseudonocardia endophytica* YIM 56035^T^	Chen et al., 2009
Arid	*Elaeagnus angustifolia*	*Micromonospora* sp. D30401, D30202, D30511 C10401 and D30407, *Nonomureae* sp. D10204, *Pseudonocardia* sp. C20201 *planotetraspora* sp. C10404	Chen et al., 2011
Arid	*Aloe arborescens*	*Micrococcus aloeverae* AE-6^T^*, Streptomyces zhaozhouensis* NEAU-LZS-5^T^	He et al., 2014; Prakash et al., 2014
Arid (Cold)	*Psammosilene tunicoides*	*Allostreptomyces psammosilenae* YIM DR4008^T^	Huang et al., 2017
Mangrove	*Centella asiatica*	*Streptomyces* sp.*, wenchangensis* 234402, *Actinoplanes brasiliensis* IFO13938*, Couchioplanes caeruleus* SCC 1014*, Gordonia otitidis* IFM 10032, *Micromonospora schwarzwaldensis* HKI0641	Ernawati et al., 2016
Mangrove	*Terminalia mucronata*	*Micromonospora terminaliae* CAP94^T^	Kaewkla et al., 2017
Mangrove	Mangrove medicinal Plants of Macao	*Friedmanniella* sp. 4Q3S-3 and *Nakamurellas* sp. 2Q3S-4-2	Li et al., 2017
**OTHER PLANTS**			
Mangroves	*Jatropha curcas*	*Jatrophihabitans endophyticus* S9-650^T^, *Nocardioides panzhihuaensis* KLBMP 1050^T^, *Nocardia endophytica* KLBMP 1256^T^*, Kibdelosporangium phytohabitans* KLBMP 1111^T^	Qin et al., 2011, 2012b, 2015; Xing et al., 2011; Madhaiyan et al., 2013
Mangrove	*Sonneratia apetala*	*Micromonospora sonneratiae* 274745^T^	Li et al., 2013
Mangrove	*Bruguiera sexangula*	*Mangrovihabitans endophyticus* S3Cf-2^T^	Liu S. W. et al., 2017
Saline	*Salicornia europaea*	*Modestobacter roseus* KLBMP 1279^T^	Qin et al., 2013a
Saline	*Tamarix chinensis*	*Streptomyces halophytocola* KLBMP 1284^T^	Qin et al., 2013b
Saline	*Dendranthema indicum*	*Glycomyces phytohabitans* KLBMP 1483^T^, *Amycolatopsis jiangsuensis* KLBMP 1262^T^	Xing et al., 2013, 2014
Saline	*Viola philippica*	*Micromonospora violae* NEAU-zh8^T^	Zhang et al., 2014b
Saline	*Costus speciosus*	*Micromonospora costi* CS1-12^T^	Thawai, 2015
Saline	*Glycyrrhiza uralensis*	*Phytoactinopolyspora endophytica*	Li et al., 2015
Saline	*Salsola affinis*	*Okibacterium endophyticum* EGI 650022^T^*, Arthrobacter endophyticus* EGI 6500322^T^	Wang et al., 2015c, Wang et al., 2015d
Arid	*Dysophylla stellata*	*Rothia endophytica* YIM 67072^T^	Xiong et al., 2013
Arid	*Anabasis elatior*	*Frigoribacterium endophyticum* EGI 6500707^T^, *Labedella endophytica* EGI 6500705^T^	Wang et al., 2015a, Wang et al., 2015b
Arid	*Anabasis aphylla* L.	*Glycomyces anabasis* EGI 6500139^T^	Zhang et al., 2018
Arid	*Ferula sinkiangensis*	*Amycolatopsis* sp. SX2R71	Liu Y. et al., 2017
Aquatic	Seaweed	*Streptomyces sp*.	Hemalatha and Rasool, 2017
Aquatic	*Thalassia hemprichii*	M*icromonospora* sp. (HCZ27 HCZ42, M8Z28, G2Z37)*, Saccharomonospora* sp. (M8Z39, G2Z41, G2Z21)*, Mycobacterium* sp. G2Z43*, Actinomycetospora lutea* G2Z35*, Nonomuraea maheshkhaliensis* M1Z44, *Verrucosispora sediminis* M1Z33, *Nocardiopsis composta* M1Z45, *Microbacterium esteraromaticum* HCZ21, *Glycomyces arizonensis* HCZ4, *Streptomyces* sp.	Wu et al., 2012

### Diversity of endophytic actinobacteria in mangrove and non-mangrove saline ecosystems

The intertidal zone of estuaries, backwaters, deltas, creeks, lagoons, marshes, and mud-lands are the main habitats of mangrove forests. They are also known as coastal wetland forests which cover around 25% of the overall world's coastline (Spalding et al., 1997; Alongi, 2002). Environment of the mangroves in terms of geographical location, pH, temperature, salinity, moisture and nutrients is highly diverse and different (Amrita et al., 2012; Xu et al., 2014). In tropical mangroves, 91% of the total microbial biomass is bacteria and fungi, another 7% is algae, and 2% is protozoa (Alongi, 1988). They are known for their productivity but very few investigations have been carried on the microbial diversity, specifically for endophytic actinobacteria.

Soil and sediments of mangrove ecosystems have been found to be excellent sources of novel actinobacterial strains (Ara et al., 2007; Han et al., 2007; Eccleston et al., 2008; Huang et al., 2008; Azman et al., 2015). While similar extensive reports are not awailable for endophytic actinobacteria but the novelity in endophytic community could be expected since soil and sedinments serve as reservoir for them. Gupta et al. (2009) reported 105 isolates of endophytic actinobacteria from 19 mangrove plants of Bhitarkanika region, India. Characterization of these isolates led to identification of 20 *Strptomyces* spp., wherein *S. exfoliates* and *S. auranticus* were reported from all the plants. But *S. halstedii, S. longisproflavus* and *S. albidoflavus* were reported only from *Kandelia candel*. Upto 118 actinobacterial strains were isolated by Wei et al. (2010) from the plant tissue sampls of Shankou Mangrove Nature Reserve, China. Majority of these isolates were *Streptomyces* spp. (37%), followed by *Micromonospora* spp. (21%). The genera *Saccharothrix* and *Nocardia* contained three isolates each, while both *Nocardiopsis* and *Lentzea* had one isolate each. Higher numbers of endophytic actinobacteria were reported from the mangrove plant *Bruguiera gymnorrhiza* of the Andaman Islands; details are yet to be investigated (Baskaran et al., 2012). Another endophytic halotolerant actinobacteria, *Saccharopolyspora dendranthemae*, was isolated from a coastal salt marsh plant at Jiangsu, China (Zhang et al., 2013). Isolation of several actinobacterial strains belonging to the genera S*treptomyces, Nocardiopsis, Pseudonocardia, Agrococcus*, and *Isoptericola* have been reported from the mangrove plants in Beilun River, Beilum Estuary National Nature Reserve, China (Yang et al., 2015). These isolates were found to produce bioactive compounds which inhibited pathogens like *Escherichia coli, Pseudomonas aeruginosa, Staphylococcus aureus, Proteus vulgaris*, hemolytic S*treptococcus* sp., and *Klebsiella pneumoniae*. A novel endophyte, *Phycicoccus endophyticus*, was reported from the *Bruguiera gymnorhiza*, a plant that belonged to Zhanjiang Mangrove Forest National Nature Reserve, Guangdong, China (Liu et al., 2016). Isolation of 11 actinobacterial strains was reported from the mangrove plants, *Rhizophora mucronata* and *Sonneratia caseolaris*. These isolates were found to be good source of antimicrobial and antioxidant compounds (Mesta et al., 2017). A large number of mangrove forests still remain untapped and could prove to be novel sources for isolation of rare and environmentally important endophytic actinobacteria.

Mangrove endophytes are rich in diversity as more than 10 isotales belonging to new species have been discovered from this source in the last few years (Jiang et al., 2017; Liu S. W. et al., 2017; Sun et al., 2017). A new species of endophytic actinobacteria belonging to the genus *Marmoricola* was reported for the first time from the mangrove plant (Jiang et al., 2017). Jiang et al. (2018) has recently reported endophytic actinobacteria from five different mangrove plants from Beilun Estuary National Nature Reserve, China. Prolific diversity of culturable endophytic actinobacteria had been reported, which included 7 orders, 15 families and 28 genera with significant novelty as there were potentially 7 new species of different genera. Furthermore, the genera, *Sanguibacter*, and *Citricoccus*, were reported for the first time in this study. Mangrove plants of Macao were also reported to be diverse in terms of novel genera and species of endophytic actinobacteria (Li et al., 2017). Two new species of rare genera *Friedmanniella* and *Nakamurella* were found among 192 endophytic actinobactrial strains isolated from 12 plants, which included 30 genera, 17 families, and 8 orders (Li et al., 2017). *Mangrovihabitans endophyticus*, a novel genus and novel species was reported from the mangrove plant *Bruguiera sexangula* (Liu S. W. et al., 2017). A large number of mangrove forests still remain untapped and could prove to be sources for isolation of rare and ecologically important endophytic actinobacteria.

Beside mangroves, halophytic plants from other saline environments have also been explored for endophytic actinobacteria. Saline conditions present a harsh environment for plant species as only <0.2% of the species are evolved to reproduce when exposed to seawater (Flowers and Colmer, 2015). Endophytes residing within such plants are reported to be one of the critical factors that contribute to the survival of these plants in saline environment (Sgroy et al., 2009). A few reports are available on the isolation and characterization of endophytic actinobacteria. A novel isolate, *Modestobacter roseus* KLBMP 1279^T^, was obtained from the roots of *Salicornia europaea*, a halophyte of coastal region of Jiangsu Province, China (Qin et al., 2013a). A novel endophytic actinobacterium, *Okibacterium endophyticum*, capable of growth at high salt concentration (upto 7% NaCl) and at high pH was reported to be inhabitant of the roots of the halophyte *Salsola affinis* (Wang et al., 2015c). *Amycolatopsis jiangsuensis* sp. nov., was isolated from a coastal plant *Dendranthema indicum* (Linn.) Des Moul collected from the coastal region of Nantong, China. From this data and some other reports summarized in Table 1, it is evident that novel actinobacterial species had been reported from the halophytic plants of various regions. A scan through literature appeared to suggest that halophytic plants presented an attractive source for novel endophytic actinobacteria, most of which are yet to be explored.

### Diversity of endophytic actinobacteria in aquatic ecosystems

The aquatic ecosystem is the largest contributor of the earth's biodiversity. Two fifths of earth's surface is freshwater ecosystem while it's 20th part of the total water (Alexander and Fairbridge, 1999). Lentic (include pools, ponds, and lakes), lotic (streams and rivers) and wetlands are the basic freshwater ecosystems. Rooted plants mainly occur in shallow water. Roots in aquatic plants are poorly developed (Sculthorpe, 1985). Association of diverse actinobacterial communities with marine sponges and soft corals has generated keen interest among researchers (Govindasamy et al., 2014). In view of these findings, it can be expected that significant diversity and novelty in endophytic actinobacterial communities of aquatic plants is waiting to be revealed. The wetlands, known for their fertility and nutrient richness, are among the most studied of the aquatic ecosystems. Rice plants from the wetlands had been explored for endophytic microbial communities as they might be associated with crop yield and also for their role in anaerobic methanogenesis causing global climate change (Bernstein et al., 2007).

A report on seagrass root-associated microorganisms suggested actinobacteria to be the first root colonizers (Jensen et al., 2007). In a study of 110 actinobacteria isolated from seagrass, ten genera of actinobacteria: *Streptomyces, Micromonospora, Saccharomonospora, Mycobacterium, Actinomycetospora, Nonomuraea, Verrucosispora, Nocardiopsis, Microbacterium*, and *Glycomyces* were reported along with four novel species (Wu et al., 2012). Twenty-one endophytic actinobacteria isolated from marine seaweed, Kovalam Beach, India, were used in generating copper nanoparticles that were active against *Klebsiella pneumoniae, Proteus mirabilis, Escherichia coli, Salmonella typhimurium, and* methicillin resistant *Staphylococcus aureus* (MRSA). The 16S rRNA analysis showed that 2 of the 21 isolates belong to the *Streptomyces* sp. (Hemalatha and Rasool, 2017). In another study of endophytic actinobacteria isolated from marine green algae *Cauler pataxifolia*, it was found that DSM3 had potential in producing antibiotic against multidrug resistant Gram-negative bacteria (Rajivgandhi et al., 2018). He also isolated *Nocardiopsis* sp. GRG1 (KT235640), an endophytic actinobacterium from brown algae. It was also active against multidrug resistant Gram-negative bacteria (Rajivgandhi et al., 2016). There are very few reports from the marine non-mangrove environments. Due to water logged condition and high salinity of oceans, the aquatic flora is under immense stress and is of immense importance due to its capacity to withstand such conditions. There is probability that the associating microorganisms could have played significant role in survival of this aquatic flora. Therefore, an extensive research on endophytic actinobacteria of marine plants is called for establishing their significance in growth and survival of the plants under such conditions.

### 16S rRNA gene sequence-based phylogenetic analysis of endophytic actinobacterial diversity in special niches

Nucleotide sequences of 16S rRNA gene of endophytic actinobacteria (Table 1) were retrieved from NCBI GenBank. Phylogenetic analyses of these sequences were performed using the software, Phylogeny.fr (Dereeper et al., 2008, 2010). Sequences were saved in FASTA format and then copied and pasted at the space provided in the online tool. The phylogenetic analyses were carried out using maximum likelihood. We have used one click mode which is an automated programme that performs step by step analysis starting from the alignment of the sequences (MUSCLE 3.8.31; Edgar, 2004), alignment refinement (Gblocks 0.91b), phylogeny (PhyML 3.1/3.0 aLRT) (Guindon and Gascuel, 2003; Anisimova and Gascuel, 2006) to the tree rendering (TreeDyn 198.3; Chevenet et al., 2006). Further, the Inkscape 0.92 was used to make any highlighting and editing in the tree for clarity of facts (www.inkscape.org). *Escherichia coli* K-12 strain DH10B was used as outgroup for this phylogenetic study.

**Figure 1:**
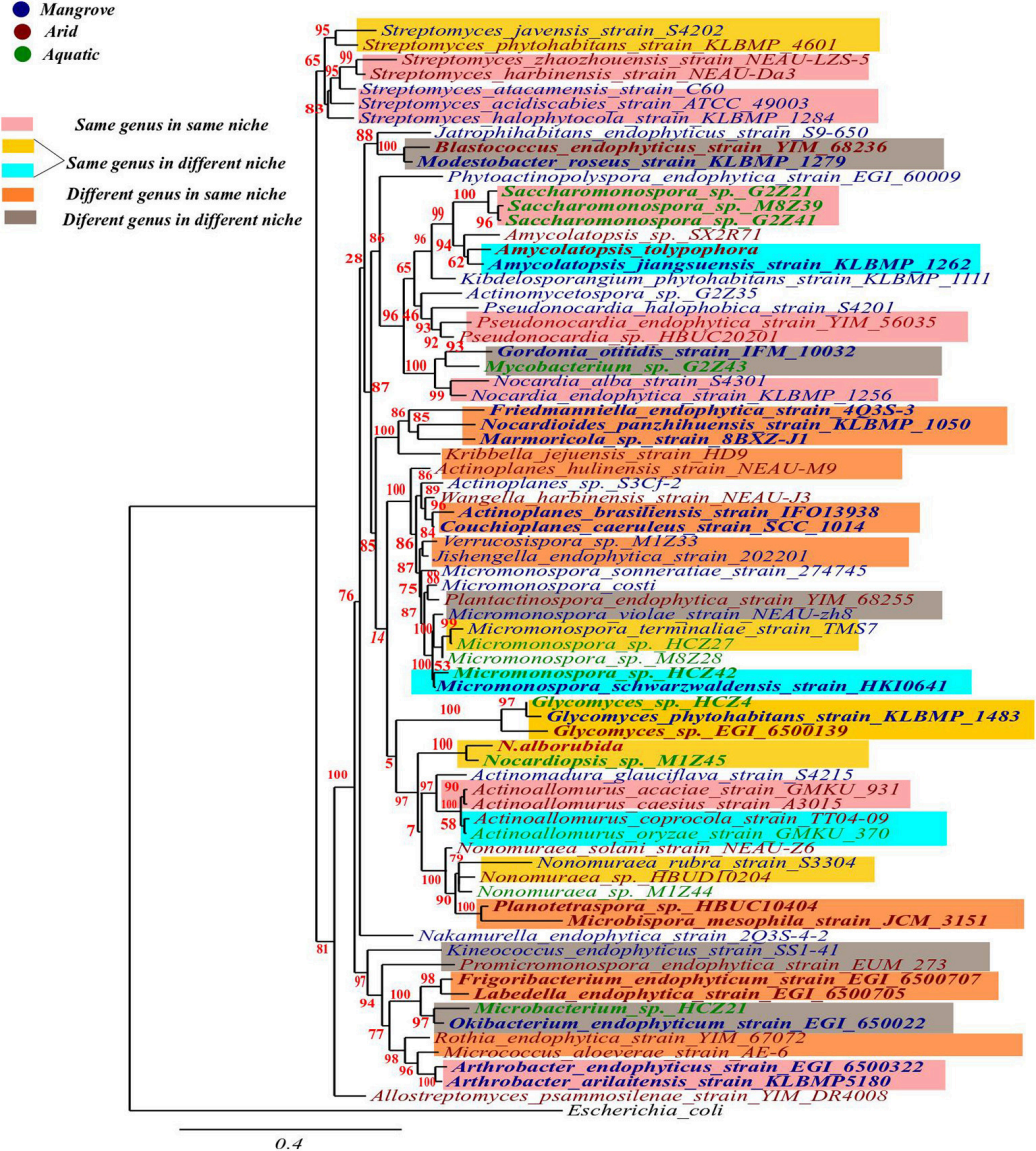
Phylogenetic diversity of some endophytic actinobacteria from extreme ecological niches based on 16S rRNA gene sequences. The tree was constructed using “one click” mode in Phylogeny.fr. *E. coli* was used as outgroup for this study.

In order to consider evolutionary relatedness among the isolates falling in the same clade, bootstrap value of ≥85% and ≥90% were considered for determining closeness among different genera and different species of the same genus respectively. Some general observations that could be made based on this criterion from the data presented in Figure 1, included: (1) Different species of the same genus either from different or the same habitats showed high bootstrap values (≥90%) indicating high degree of relatedness among them; (2) different genus from the same or different niche displayed bootstrap values of ≥85%, suggesting closer evolutionary relationship among them. In addition, different species of a genus from the same habitat were found to be placed within the same clade but the bootstrap values were <65%, indicating low confidence level in respect of their evolutionary closeness. However, for a deeper insight into evolutionary aspects of these microbes, further investigations involving other evolutionary parameters appear necessary.

## Metagenomics based diversity of endophytic actinobacteria

Culturable approach to study diversity is often limited by access to only a minor fraction of the microbial community. The metagenomic approach, which employs PCR-based culture-independent method, offers a more comprehensive view of the diversity as it includes both culturable and non-culturable part of the population. Some of the recent metagenomic studies that highlighted the importance of the uncultured endophytic actinobacteria in the diversity of endophytic actinobacterial population are being discussed.

In the study of community structure of endophytic actinobacteria of the *Pseudowintera colorata* (Horopito), a native medicinal plant of New Zealand, it was seen that the microbial communities in leaves were more diverse as compared to the roots and stems on the basis of DGGE pattern. The abundance of actinobacterial taxa was highest in stems (39%), followed by leaves (34%), and roots (27%). However, three clones among them were identified as uncultured bacteria (Purushotham et al., 2018). Similar study of the endophytic actinobacterial population from medicinal plant *Centella asiatica* produced 16 major clones pertaining to the various communities of actinobacteria in the sample. Community of actinobacteria in the plant tissues was slightly more diverse than those of rhizosphere in this case (Ernawati et al., 2016). However, *Streptomyces* sp. was the abundant followed by members of the family *Micromonosporaceae* and *Gordoniaceae*. Furthrmore, the uncultured bacterial abundance was also significantly high. It shows that there is a need for optimization of isolation procedures in order to enhance the chances (Ernawati et al., 2016). According to a report on endophytic actinobacterial species diversity in the stem of *Gynura cusimbua* by 16S rRNA, it was observed that out of 63 positive clones, 16S rRNA sequence of 59 strains had higher similarity to the closest type strain and belonged to the genera *Microbacterium, Arthrobacter, Micrococcus, Curtobacterium, Okibacterium, Quadrisphaera, and Kineococcus* respectively (Zhang et al., 2016). On the other hand, the rest of clones were showing little similarity and belonged to unclassified families: *Micrococcineae, Intrasporangiaceae*, and *Microbacteriaceae* that are supposed to be uncultured endopohytic actinobacteria (Zhang et al., 2016). Culture independent methods were also used for the community study of the endophytes related to the sponges, *Hymeniacidon perleve* and *Sponge* sp. An uncultured actinobacterium of the genus *Acidimicrobium* accounted for 33% and 24%, respectively in the above two sponges (Xin et al., 2008). Some other reports are also available on the metagenomic studies of stem and roots of rice (Tian et al., 2007), roots of wheat (Conn and Franco, 2004), roots and shoots of *Aquilaria crassna* (Nimnoi et al., 2010), leaves of grapevine (Bulgari et al., 2009), and *Maytenus austroyunnanensis* (Qin et al., 2012a). These studies revealed that the diversity of uncultured endophytic actinobacteria is comparable to those of the cultured endophytic actinobacteria. In order to get complete information on the diversity of endophytic actinobacteria and their functional importance, it is important to study the uncultured endophytic actinobacteria by metagenomic approach along with the culturable ones.

## Chemical diversity and therapeutic significance of secondary metabolites produced by endophytic actinobacteria

Phytochemical profiling to identify and discover therapeutic compounds from plant extracts has been a common practice since long (Tiwari et al., 2011; Ahmad et al., 2013). However, comparable studies on microbial extracts are scanty. In view of immense potential of the secondary metabolites of actinobacteria for diverse applications, it is highly desirable that vigourous efforts are made to explore the chemical diversity of these metabolites. It is in the light of this observation that the relevant information from literature is being presented here.

Actinobacteria are known producers of a broad spectrum of bioactive molecules. They employ non-ribosomal peptide synthetase (NRPS) and polyketide synthase (PKS) pathways to achieve the structural and functional diversity of their secondary metabolites. They are reported to produce immunosuppressants (e.g., rapamycin), antibiotics (e.g., erythromycin), anticholesterol drugs (e.g., lovastatin), and anticancer drugs (e.g., epothilone B) which underlined their indispensibility for the pharmaceutical industries (Miller et al., 2012; Weber et al., 2015). Actinobacteria in general and *Streptomyces* spp. in particular have been explored extensively for drugs and therapeutics. The secondary metabolites produced by the endophytic actinobacteria were reported to fall majorly under pharmaceutically important classes like alkaloids, flavonoids, steroids, terpenoids, phenolics, quinones, and peptides (Yu et al., 2010). For instance, in a study of isolation and characterization of compounds from mangrove endophytic actinobacteria, 73 novel compounds and 49 known compounds were characterized as alkaloids, benzene derivatives, cyclopentenone derivatives, dilactones, macrolides, 2-pyranones and sesquiterpenes (Xu et al., 2014). Chemical diversity of therapeutically important metabolites from endophytic actinobacteria have been analyzed in the present work. Chemical classes have been defined for several of such compounds, as presented in Table 2 and discussed briefly in the following sections. Strucutre of some of these compounds has been given in Table 3.

**Table 2:** Chemical diversity of the metabolites of endophytic actinobacteria and their therapeutic significance.

**Bioactive Compounds**	**Habitat**	**Plant source**	**Therapeutic application**	**Actinobacteria species**	**References**
**ALKALOIDS**					
6-Prenylindole	Temperate	*Allium tuberosum*	Antifungal	*Streptomyces* sp. TP-A0595	Sasaki et al., 2002
Anicemycin	Temperate	*Aucuba japonica*	Antitumor (anchorage-independent growth inhibitor)	*Streptomyces thermoviolaceus* TP-A0648	Igarashi, 2004
Kakadumycin A	Arid	*Gravillea pteridifolia*	Antibacterial, Antimalaria	*Streptomyces* sp. NRRL 30566	Castillo et al., 2003
Diketopiperazine, Gancidin W	Tropical	*Shorea ovalis*	Low toxic, Antimalarial agent	*Streptomyces* sp. SUK10	Zin et al., 2017
Cyclo-(L-Val-L-Pro), Cyclo-(L-Leu-L-Pro), Cyclo-(L-Phe-L-Pro), Cyclo-(L-Val-L-Phe), and N-(7-hydroxy-6-methyl-octyl)-acetamide. (Diketopiperazines)	Tropical	*Zingiber spectabile*	Antibacterial	*Streptomyces* sp.SUK 25	Alshaibani et al., 2017
1-Acetyl-β-carboline, Indole-3-carbaldehyde, 3-(Hydroxyacetyl)-Indole, Brevianamide F, and Cyclo-(L-Pro-L-Phe)	Aquatic (wetland)	*Vochysia divergens*	Antibacterial	*Aeromicrobium ponti* LGMB491	Gos et al., 2017
1-Vinyl-b-carboline-3-Carboxylic acid, Indole-3- carbaldehyde, Indole-3-acetic acid and Indole-3- carboxylic acid	Aquatic (wetland)	*Vochysia divergens*	Antibacterial activity	*Microbispora* sp. LGMB259	Savi et al., 2015b
Lansai A-D	Tropical	*Ficus benzamina*	Antifungal and anticancer	*Streptomyces* sp. SUC1	Tuntiwachwuttikul et al., 2008
3-Acetonylidene-7-Prenylindolin-2-one and 7-Isoprenylindole-3-carboxylic acid	Arid (Cold)	*Glycine max*	Antifungal	*Streptomyces* sp. neau-D50	Zhang et al., 2014a
2-(furan-2-yl)-6-(2S,3S,4-trihydroxybutyl)pyrazine	Mangrove	*Xylocarpus granatum*	Antiviral	*Jishengella endophytica* 161111	Wang et al., 2014
**POLYKETIDES**					
Pterocidin	Subtropical	*Pteridium aquilinum*	Anticancer	*Streptomyces hygroscopicus* TP-A0451	Igarashi et al., 2006
Linfuranone	Tropical	*Clinacanthus siamensis*	Antimicrobial, non-cytotoxic	*Microbispora* sp. GMKU 363	Indananda et al., 2013
Clethramycin	Tropical	*Clethra barbinervis*	Antifungal	*Streptomyces hygroscopicus* TP-A0623	Furumai et al., 2003
Ansamitocin	Tropical	*Maytenus serrata*	Antibacterial and antitumor	*Actinosynnema pretiosum*	Higashide et al., 1977; Siyu-Mao et al., 2013
**TERPENES AND TERPENOIDS**					
Demethylnovobiocins	Temperate	*Aucuba japonica*	Antimicrobial	*Streptomyces* sp. TPA0556	Igarashi, 2004
Cedarmycin A and B	Temperate	*Aucuba japonica*	Antibacterial, anti-Candida	*Streptomyces* sp. TP-A0456	Sasaki et al., 2001
Xiamycin	Mangrove	*Bruguiera gymnorrhiza*	Antiviral	*Streptomyces* sp.GT2002/1503	Ding et al., 2010
**COUMARINS (ALPHA BENZOPYRONES)**					
5,7-Dimethoxy-4-pmethoxylphenylcoumarin; 5,7-Dimethoxy-4-phenylcoumarin	Tropical	*Zingiber officinale*	Antifungal agent, Antioxidants, Antitumor	*Streptomyces aureofaciens* CMUAc130	Taechowisan et al., 2007
Saadamycin	Marine	*Aplysina fistularis*	Antifungal agent	*Streptomyces* sp. Hedaya 48	El-Gendy and El-Bondkly, 2010
**FLAVONOIDS (GAMMA BENZOPYRONES)**					
7-Methoxy-3, 3′,4′,6-tetrahydroxyflavone and 2′,7-Dihydroxy-4′,5′-Dimethoxyisoflavone, Fisetin, Naringenin, 3′-Hydroxydaidzein, Xenognosin	Tropical	*Boesenbergia rotunda (L.)*	Antibacterial	*Streptomyces* sp. BT01	Taechowisan et al., 2014
Kaempferol, Isoscutellarin, Umbelliferone and Cichoriin	Temperate	*Alpinia galanga*	Antioxidants	*Streptomyces* sp. Tc052	Taechowisan et al., 2009
**QUINONES**					
Alnumycin	Temperate	*Alnus glutinosa*	Antibacterial	*Streptomyces* sp.DSM 1175	Bieber et al., 1998
Celastramycins A and B	Tropical	*Celestracae* family plants	Antimycobacterial, Antibacterial	*Streptomyces setonii*, sp. Q21, *Streptomyces sampsonii*, QuH- 8	Pullen et al., 2002
Lupinacidin C	Arid	*Lupinus angustifolius*	Antitumor	*Micromonospora lupini* Lupac 08	Trujillo et al., 2007; Igarashi et al., 2011
Naphtomycin A	Tropical	*Maytenus hookeri*	Antitumor	*Streptomyces* sp. CS	Lu and Shen, 2007
**TANNINS**					
Streptol	Arid (Cold)	*Cucubalus* sp.	Anti-fungal	*Dactylosporangium*sp. *strain* SANK 61299	Okazaki, 2003
**PEPTIDES AND THEIR DERIVATIVES**					
Actinomycin X2	Temperate	*Rhododendron* sp.	Antimicrobial	*Streptomyces* sp. R-5	Shimizu et al., 2004
Munumbicins A, B, C and D	Tropical	*Kennedia nigricans*	Antimicrobial, Antimalarial, Antitumor	*Streptomyces* sp. NRRL 30562	Castillo et al., 2002
Coronamycin	Tropical	*Monstera* sp.	Antifungal, Antimalarial	*Streptomyces*. sp. MSU-2110	Ezra et al., 2004
Munumbicins E-4 and E-5	Tropical	*Kennedia nigricans*	Antimalarial, antibacterial	*Streptomyces* sp. NRRL 30562	Castillo et al., 2006
S-adenosyl-Nacetylhomocysteine	Tropical	*Puereria candollei*	Antioxidant, Neuroprotection	*Micromonospora* sp. PC1052.	Boonsnongcheep et al., 2017
Proximicin	Arid	*Sonchus oleraceus*	Antibacterial, Antitumor	*Verrucosispora maris* AB-18-032.	Fiedler et al., 2008; Roh et al., 2011; Ma et al., 2016
**FATTY ACID DERIVATIVE**					
6-alkalysalicilic acids, salaceyins A and B	Arid	*Ageratum conyzoides*	Anticancer	*Streptomyces laceyi* MS53	Kim et al., 2006
7-Octadecenamide	Arid	*Sonchus oleraceus*	Antimicrobial	*Nocardia caishijiensis*	Tanvir et al., 2016
9, 12- Octadecadienamide (Linoleamide)	Arid	*Sonchus oleraceus*	Antimicrobial	*Pseudonocardia carboxydivorans* SORS 64b	Tanvir et al., 2016

**Table 3:** Chemical Structures of some important therapeutic compounds of different chemical classes isolated from endophytic actinobacteria.

**Bioactive compounds**	**Chemical structure**	**References**
**ALKALOIDS**		
6 Prenylindole	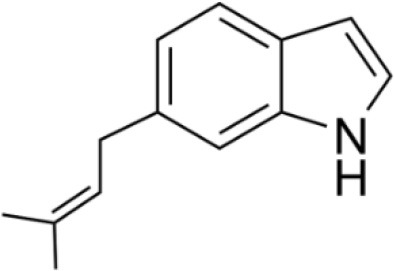	Sasaki et al., 2002
Anicemycin	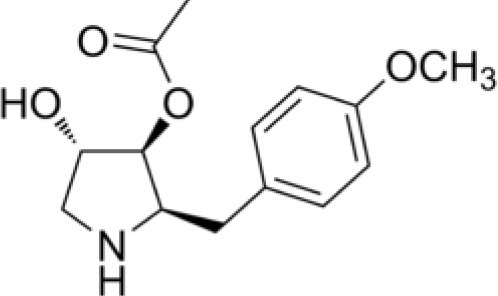	Igarashi, 2004
Gancidin W	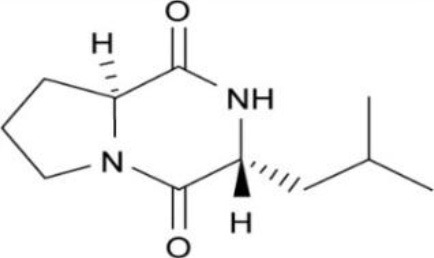	Zin et al., 2017
Brevinamide F	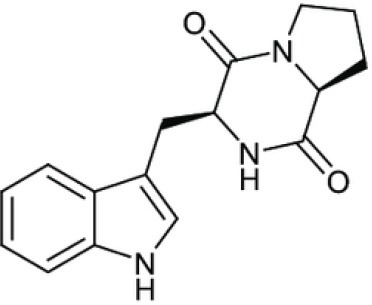	Amar et al., 2012
Lansai A; R = H, Lansai B; R = CH_3_	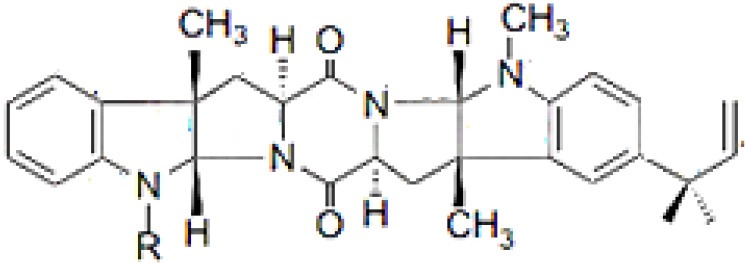	Tuntiwachwuttikul et al., 2008
Lansai C; R = OH, D; R = H	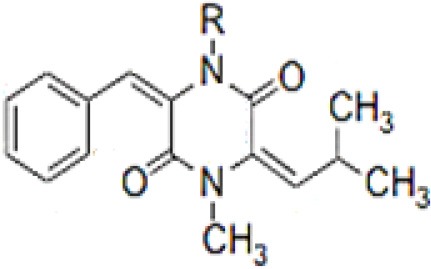	Tuntiwachwuttikul et al., 2008
2-(furan-2-yl)-6-(2S,3S,4-trihydroxybutyl)pyrazine R = 1 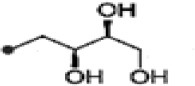 R2 = H	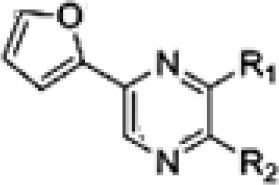	Wang et al., 2014
**POLYKETIDES**		
Pterocidin	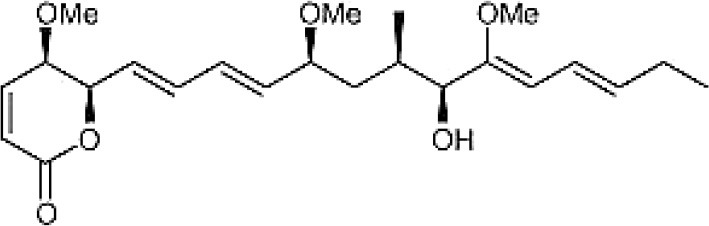	Igarashi et al., 2006
Linfuranone	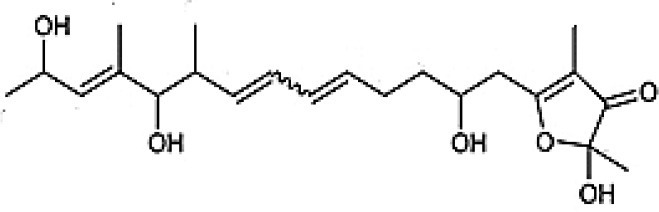	Indananda et al., 2013
Ansamitocin	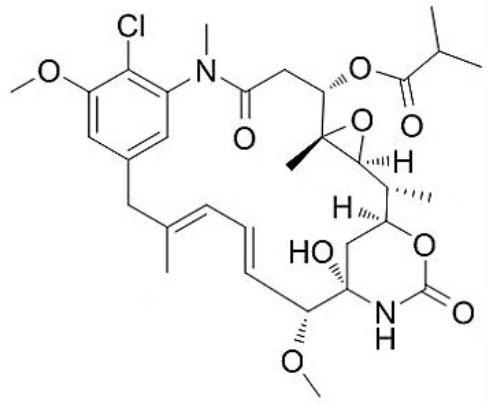	Siyu-Mao et al., 2013
**TERPENES AND TERPENOIDS**		
Demethylnovobiocins	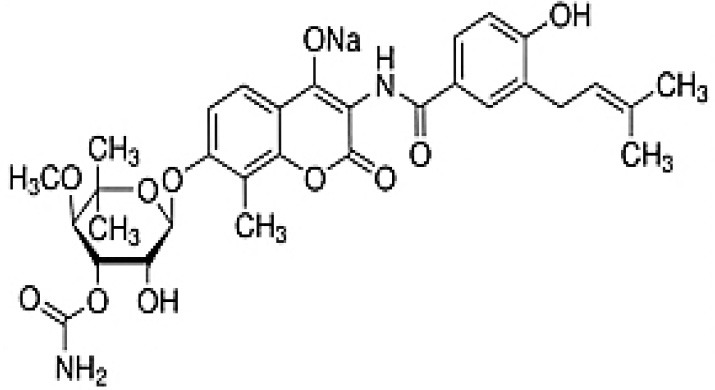	Kominek, 1972
Cedarmycin	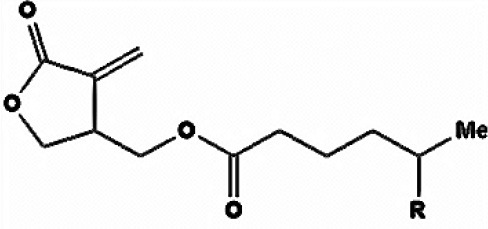	Sasaki et al., 2001
Xiamycin	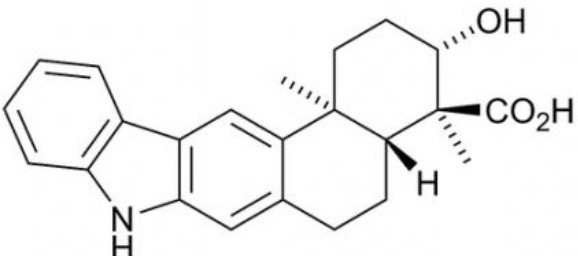	Li et al., 2012
**COUMARINS (ALPHA BENZOPYRONES)**		
5,7-Dimethoxy-4- pmethoxylphenylcoumarin	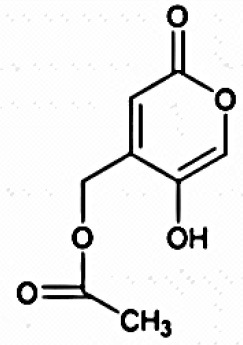	Indraningrat et al., 2016
Saadamycin	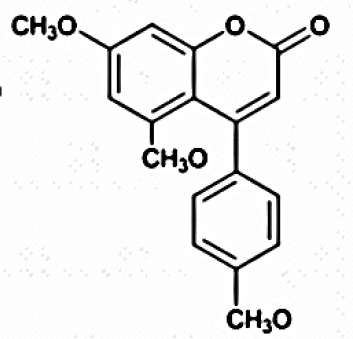	Indraningrat et al., 2016
**FLAVONOIDS (GAMMA BENZOPYRONES)**		
7-Methoxy-3, 3′,4′,6-tetrahydroxyflavone	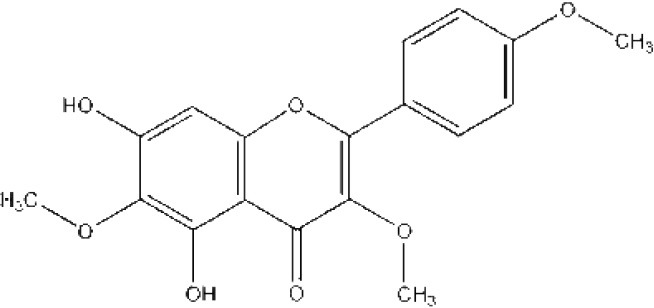	Molinstincts chemical structures ID CT1105232405
Fisetin	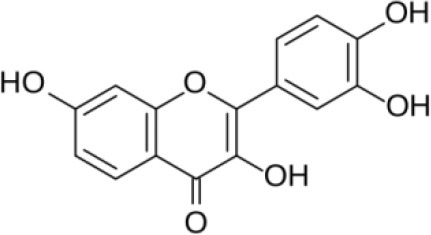	Khan et al., 2013
Naringenin	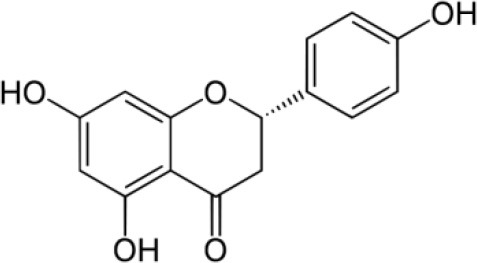	Taechowisan et al., 2014
Xenognosin	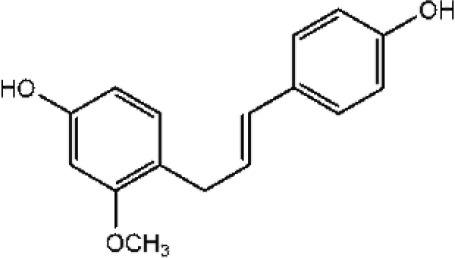	Taechowisan et al., 2014
Hydroxydaidzein	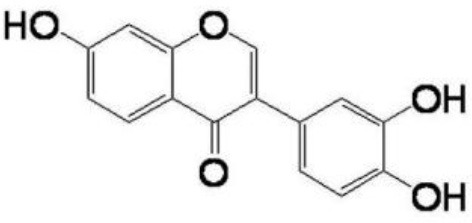	Taechowisan et al., 2014
Alnumycin	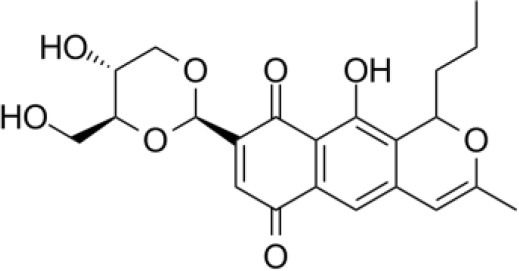	Bieber et al., 1998
**QUINONES**		
Celestamycin A	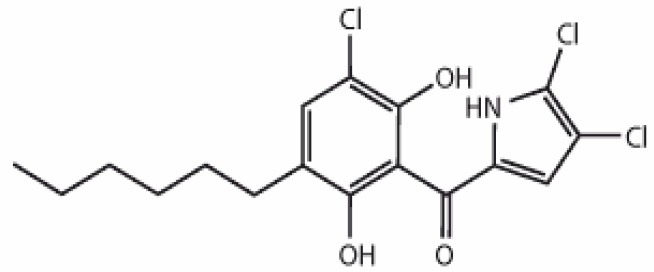	Pullen et al., 2002
Celestamycin B	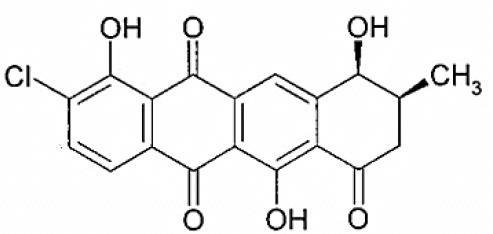	Pullen et al., 2002
Lupinacidin (R = CH3 or H)	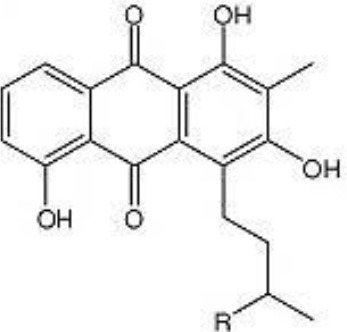	Igarashi et al., 2011
Naphthomycin A	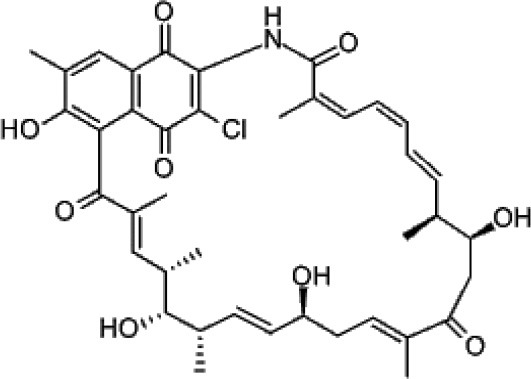	Lu and Shen, 2007
**TANNINS**		
Streptol	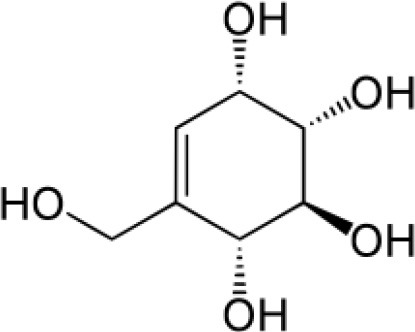	Chemspider ID 4450703
**PEPTIDES AND THEIR DERIVATIVES**		
Actinomycin X2	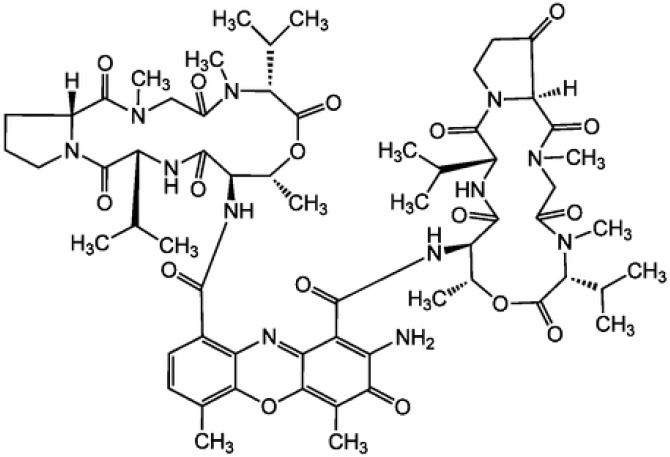	Shimizu et al., 2004
S-adenosyl-N acetylhomocysteine	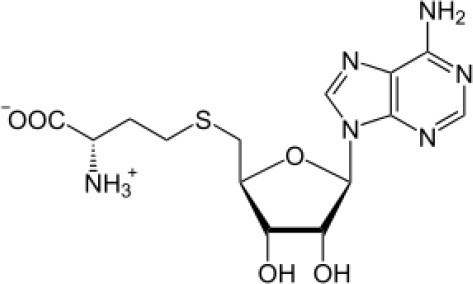	Boonsnongcheep et al., 2017
Proximicin A	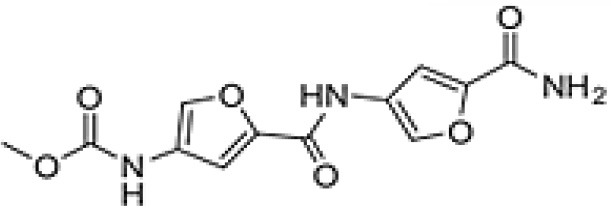	Fiedler et al., 2008
**FATTY ACID DERIVATIVE**		
Linoleamide	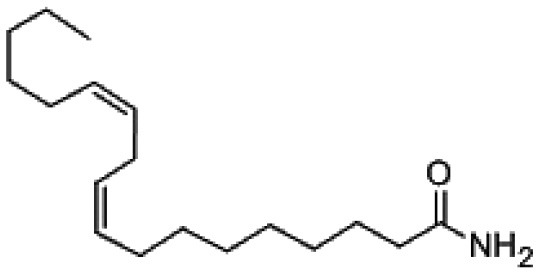	CAS:3072-13-7
Salaceyins A(R = CH_3_) ; B (R = CH_2_CH_3_)	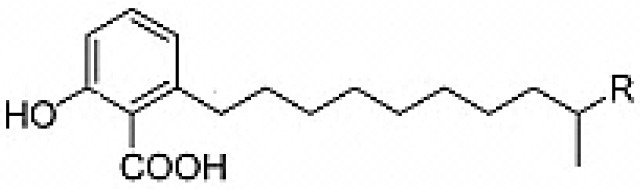	Kim et al., 2006

### Alkaloids

Alkaloids are characterized by the presence of basic nitrogen (usually in a heterocycle). In recent years, the number of new alkaloids isolated, identified, and characterized for their medicinal potential from various sources including from the endophytic actinobacteria has increased. Paclitaxel (taxol), a well known anti-cancer drug, was originally isolated from the plant, *Taxus baccata*. Subsequently, it was also isolated from the endophytic actinobacteria of *Taxus baccata*: *Kitasatospora* sp. P and U 22869 (Caruso et al., 2000). Recently, various alkaloids, namely,1-acetyl-β-carboline, indole-3-carbaldehyde, tryptophol, 3-(hydroxyacetyl)-indole, brevianamide F, cyclo-(L-Pro-L-Phe), cyclo-(L-Pro-L-Tyr), cyclo-(L-Pro-L-Leu), and cyclo-(L-Val-L-Phe) were reported from the endophyte, *Aeromicrobium ponti* LGMB491 isolated from *Vochysia divergens* (Gos et al., 2017). These compounds displayed very good antibacterial activities against various *Staphylococcus* spp. Other alkaloids, 3-acetonylidene-7-prenylindolin-2-one, and 7-isoprenyl indole-3-carboxylic acid, isolated from endophytic *Streptomyces* sp. neau- D50, possessed anti-cancer activity (Zhang et al., 2014a). β-Carbolines and 3-Indole compounds were obtained from the endophyte *Microbiospora* sp. LGMB259, which showed inhibitory activities against Gram-positive bacterial strains: *Micrococcus luteus* NRRL B-2618 and *Kocuria rosea* B-1106. They aslo possess cytotoxic activities against two human cancer cell lines: prostate cancer cell line PC3 and the non-small-cell lung carcinoma cell line (Savi et al., 2015a). Diketopiperazine also belongs to this family with very good medicinal property. In this context the antifungal and anticancer compounds, Lansai A-D, were reported from the endophytic *Streptomyces* sp. SUC1 isolated from aerial roots of *Ficus benjamina* (Tuntiwachwuttikul et al., 2008). Diketopiperazine gancidin W (GW) was isolated from the *Streptomyces* sp. SUK10, an endophyte from the bark of *Shorea ovalis* tree, and it was tested *in vivo* against *Plasmodium berghei* PZZ1/100 (Zin et al., 2017). Kakadumycin A, a quinoxaline antibiotic with broad spectrum activity against Gram-positive bacteria and malaria parasites was reported from endophytic *Streptomyces* sp. NRRL 30566, isolated from the fern, *Grevillea pteridifolia*. It was structurally similar to echinomycin, but had shown better antibiotic activity (Castillo et al., 2003). Antifungal compound, 6-Prenylindole was isolated from *Streptomyces* sp. TP-A0595, an endophyte of liverwort (Sasaki et al., 2002). Igarashi (2004) isolated pure compound anicemycin, a cytotoxic compound potentially active against tumor cell line at very low concentration.

### Polyketides

This group of actinobacterial secondary metabolites includes compounds with diverse chemical structures having significant activities of therapeutic importance, like, anticancer, antifungal, anticholesteremic etc. Many reports have shown that endophytic actinobacteria are proven to be good source for the discovery of polyketide compounds. Endophyte *S. hygroscopicus* TP-A0623, isolated from the root of *Clethra barbinervis*, produced a polyene polyketide, clethramycin, known to inhibit both spheroplast regeneration and germ tube formation in *Candida albicans*, (Furumai et al., 2003). Ansamitocin, a polyketide macrolide, was isolated from endophytic actinobacteria *Nocardia* sp. no. C-15003 (Higashide et al., 1977). It had potent antibacterial and antitumor activities, and was structurally similar to maytansine, a known antibacterial drug. Another drug, pterocidin, derived from endophytic isolate, *S. hygroscopicus* TP-A0451 was cytotoxic against some human cancer cell lines (Igarashi et al., 2006). The compound, linfuranone isolated from Thai medicinal plant has shown antidiabetic and antiatherogenic activities in mouse ST-13 pre-adiopocytes (Indananda et al., 2013). Several polyketides compounds of endophytic actinobacterial origin were reported as summarized in Table 2.

### Terpenes and terpenoids

Terpenes and terpenoids are known to be the primary constituents of the essential oils of many types of medicinal plants and flowers. They have been widely used for fragrances in perfumery, and in medicines. Besides plants, microbes have also been proved to be good sources for such compounds (Tholl, 2015). Biologically active terpenes and terpenoids have been isolated from several endophytic actinobacteria (Table 2). Cedarmycin A and B, isolated from an endophyte, *Streptomyces* sp. TPA0456, were found to possess broad spectrum anti-bacterial, and anti-fungal activities (Sasaki et al., 2001). It was seen to be specifically more potent against *Candida glabrata*. Only a few compounds with anti-viral activity had been reported from endophytic actinobacteria. One among them being xiamycin, produced by *Streptomyces* sp. GT2002/1503, isolated from *Bruguiera gymnorrhiza* that specifically blocked R5 tropic HIV infection (Ding et al., 2010).

### Benzopyrones

The compounds from this class consist of a benzene ring joined to a pyrone ring. The benzopyrones can be subdivided into the benzo-alpha pyrones, which include mainly coumarins and the benzo-gamma pyrones, of which the flavonoids are principal members (Jain and Joshi, 2012).

#### Coumarins

(Benzo-alpha pyrones): Several novel compounds isolated from the endophytic actinobacteria belong to this class. *Streptomyces* sp. TPA0556 isolated from the plant *Aucuba japonica* produced two novel antibiotics: 7′- demethylnovobiocin and 5″-demethylnovobiocin with broad spectrum antibiotic activity against both Gram-positive and Gram-negative bacterial pathogens (Sasaki et al., 2001). A novel coumarin, saadamycin was reported from *Streptomyces* sp. Hedaya48 with significant antimycotic activity specifically against dermatophytes (El-Gendy and El-Bondkly, 2010). 5, 7-Dimethoxy-4-phenylcoumarin extracted from endophytic *S. aureofaciens* was effective in preventing or delaying formation of metastases (Taechowisan et al., 2007).

#### Flavonoids

(Gamma-benzopyrones): Flavonoids are polyphenolic structures, widely found in fruits, vegetables and certain beverages. They are known for their biochemical and antioxidant effects associated with various diseases such as cancer, Alzheimer's disease (AD), atherosclerosis etc. (Panche et al., 2016). Some of the flavonoids from endophytic actinobacteria include: 7-methoxy-3,3′,4′,6-tetrahydroxyflavone; 2′,7-dihydroxy-4′,5′-dimethoxyisoflavone, fisetin, naringenin, 3′-hydroxydaidzein and xenognosin B. They were isolated from endophytic *Streptomyces* sp. BT01. It has shown good activity against Gram-positive bacteria; *Staphylococcus aureus* ATCC25932, *Bacillus cereus* ATCC 7064, and *Bacillus subtilis* ATCC 6633 (Taechowisan et al., 2014). Kaempferol, isoscutellarin, umbelliferone, and cichoriin are some other flavonoids obtained from the endophytic *Streptomyces* sp. Tc052, which showed anti-oxidative and inhibitory activities on nitric oxide production (Taechowisan et al., 2009).

### Tannins

They are water soluble polyphenols that are majorly found in plant foods. They are reported to be involved in improving human health by providing anticancer and antibacterial activities. However, in many cases they can be inhibitory as they decrease the nutritional value of foods by forming complexes with proteins, starch, and digestive enzymes (Chung et al., 1998). Not many compounds of tannins family are reported from endophytic actinobacteria. Streptol, a tannin isolated from endophytic strain SANK 61299 of the actinobacterium *Dactylosporangium* sp. from the plant, *Cucubalus* sp. exibited broad spectrum antibacterial activity against both Gram-positive and Gram-negative bacteria (Furumai et al., 2003).

### Quinones

Quinones are a group of compounds produced by plants, fungi, and bacteria. They have an aromatic di-one or di-ketone system, generally derived from the oxidation of hydroquinones. They show potential therapeutic activities like neurological, antibacterial, antiplasmodial, antioxidantal, trypanocidal, antitumor, and anti-HIV that are shown to be related to the redox properties of their carbonyl functions (Villamil et al., 2004).

*Streptomyces* sp. CS, isolated from *Maytenus hookeri*, came into focus for the production of naphthomycin K, together with two known naphthomycins A and E which had shown cytotoxicity against P388 and A-549 cell lines (Lu and Shen, 2007). Another naphthoquinone, alnumycin was isolated from *Streptomyces* sp. DSM 11575, an endophyte inhabiting the root nodules of *Alnus glutinosa*. It has displayed narrow spectrum antibacterial activity against *Bacillus subtilis* tested by well diffusion assay. Endophytic actinobacterium *Micromonospora lupine* had been reported to produce anticancer compound lupinacidin (anthraquinone) A-C. Lupinacidin C was shown to inhibit invasion of murine colon carcinoma cells into the reconstituted basement membrane (Igarashi et al., 2011). Endophytic *Streptomyces* sp. MaBQuH-8, isolated from woods of the trees of family *Celastraceae*, was found to produce antibiotics: celastramycins A and B having antimicrobial activity against Gram positive bacteria and mycobacteria.

### Fatty acid derivatives

Some bioactive compounds reported from endophytic actinobacteria fall into the category of fatty acid derivatives. The compounds: 7-coctadecenamide and 9,12-octadecadienamide (Linoleamide), were derived from two rare actinobacteria, *Nocardia caishijiensis* and *Pseudonocardia carboxydivorans*, isolated from the plants *Sonchus oleraceus* and *Ageratum conyzoides* respectively. These compounds had shown very good activity against various human pathogens including *Candida* spp. strains, Gram-positive and Gram-negative bacteria (Tanvir et al., 2016). Kim et al. (2006) studied cytotoxic activity of 6-alkalysalicilic acids, salaceyins A and B from *S. laceyi* MS53 against human breast cancer cell line.

### Peptides and their derivatives

Microorganisms play a significant role in the production of peptide antibiotics. Endophytic actinobacteria being unique in their habitat can be a source of some novel therapeutic peptides.

*Streptomyces* sp. NRRL 30562, an endophyte isolated from *Kennedia nigriscans* was reported to produce actinomycin X2, a broad-spectrum polypeptide antibiotic having potential activity against human and plant pathogenic bacteria and fungi (Castillo et al., 2006). Another endophyte, *Streptomyces* sp. TC022, isolated from the roots of *Alpinia galangal*, is capable of producing actinomycin D, highly potential as antifungal, and antitumor polypeptide. It was also active against plant and human fungal pathogens, for example, *Colletotrichum musae* and *Candida albicans* (Taechowisan et al., 2006). Coronamycin, identified as a complex of novel peptides antibiotics, was obtained from verticillate *Streptomyces* sp. MSU-2110 isolated from *Monstera* sp. It has potential activity against pythiaceous fungi and the human fungal pathogen *Cryptococcus neoformans*. It was also reported to be active against *Plasmodium falciparum* with IC_50_ value of 9.0 ng/ml. In another study on the secondary metabolites of endophytic actinobacteria isolated from roots and root nodules of *Pueraria candollei* Graham ex Benth, isolation of S-adenosyl-N-acetylhomocysteine (an antibacterial and antioxidant compound) was isolated from *Micromonospora* sp. for the first time (Boonsnongcheep et al., 2017).

### Therapeutic spectrum of uncharacterized metabolites

There are several reports demonstrating wide range of therapeutic properties for the metabolite preparations from endophytic actinobacteria. But isolation and characterization of the active compounds have not been undertaken for many of them. Endophytic *Streptomyces* sp. SUK 06, isolated from the plant *Thottea grandiflora*, was reported to produce metabolites with strong activity against bacterial pathogens including MRSA as well as phytopathogens (Ghadin et al., 2008). In a study on the endophytes associated with medicinal plants, many actinobacteria producing compounds with antibacterial activity against a panel of Gram positive and Gram negative bacterial pathogens were reported (Li et al., 2008). Like-wise endophytic actinobacteria isolated from the roots of *Alpinia galangal, Streptomyces* sp. Tc052, was shown to possess antimicrobial activity with MIC values lying between 64-128 μg/ml (Taechowisan et al., 2008). *Streptomyces* sp. TQR12-4, an endophyte of *Citrus nobilis* Lour was reported to produce thermostable antifungal compound with MIC values of 100 μg/ml and 400 μg/ml (Thao et al., 2016). Crude extracts of the metabolites from *Microbispora* sp. LGMB259, LGMB250, LGMB255 and LGMB256 showed more than 98% antitumor activity against Glioblastoma multiforme cells (Savi et al., 2015b). Furtermore, the metabolites of actinobacteria strains: SORS64b, SORS124, AGRS16, AGLS2, and AGRS19 isolated from the plants of *Asteraceae* family showed antioxidant property using DPPH assay (Tanvir et al., 2014). In another study on five endophytic isolates, it was reported that their metabolites displayed excellent antioxidant properties and were found to increase the survival of plants under oxidative stress (Babu et al., 2014). Ethyl acetate extract of cell-free broth of actinobacteria isolated from *Catharantus roseus* showed marked α-glucosidase inhibitory activity (Yokose et al., 1983). Some *in vivo* studies also showed that the partially purified metabolite preparations of endophytic actinobacterium, *Streptomyces* sp. worked effectively against diabetes by reducing systemic glucose levels in the experimental animals (Kulkarni-Almeida et al., 2011). Remarkable α-glucosidase inhibitory activity was reported by the metabolites produced by several strains of *Streptomyces* sp. isolated from the roots of Indonesian medicinal plants, *Caesalpinia sappan* (Pujiyanto et al., 2012). Antidiabetic activity was studied in the extract of *Streptomyces* sp. JQ92617, isolated from *Rauwofia densiflora*, while crude extract of *S. longisporoflavus* JX96594 was found to be a potential α-amylase and α-glucosidase inhibitor (Akshatha et al., 2014).

The foregoing discussion thus presents a strong evidence for the endophytic actinobacteria to be an excellent source for the production of a wide range of natural products with significant potential for therapeutic applications. The diversity of the compounds produced could be comparable to that of plants. The use of endophytic actinobacteria for production of therapeutic compounds may offer some distinct advantage over plants in terms of production under controlled environmental conditions for strict adherence to quality, shorter production period and sustainability.

## Biosynthetic gene clusters in endophytic actinobacteria

It is evident from the literature that the genes involved in the synthesis of bioactive secondary metabolites are present in the actinobacterial genome in the form of gene clusters (Doroghazi and Metcalf, 2013). Genome mining tools have made it more convenient to look for innovations in natural product discovery (Corre and Challis, 2007). Majority of the bioactive compounds are encoded by large gene clusters such as polyketide synthases (PKS) and non-ribosomal peptide synthetases (NRPS) with highly repetitive modules (Jackson et al., 2018).

The biosynthetic pathway of polyketides is governed by a complex enzyme system, called polyketide synthase encoded by PKS gene cluster. Aromatic polyketide synthases are encoded by PKS II. On the other hand, the polyketides formed as a result of condensation of acetate, propionate and butyrate acyl units are synthesized by modular polyketide synthase that is encoded by PKS I gene (Shinwari et al., 2013). Other than polyketide synthases, there is another class of enzyme known as Non-ribosomal peptide synthetases (NRPS). NRPS gene clusters are involved in the biosynthesis of wide range of peptide antibiotics, toxins, siderophore, anti-inflammatory compounds, immunosuupressants etc. (Cane and Walsh, 1999; Crosa and Walsh, 2002; Mansson et al., 2011). Another gene cluster ANSA (ansamicin) is involved in the production of ansamycins; while OxyB codes for glycopeptide antibiotics (Ayuso-Sacido and Genilloud, 2005). In a study on taxonomic diversity and metabolic activity of endophytic actinobacteria of some medicinal plants, it was reported that all the 80 bioactive isolates possessed atleast one test gene. The frequency of occurrence biosynthetic gene clustures in the isolates was reported to be 55.0, 58.8, 90.0, 18.8, and 8.8% for the PKS-I, PKS-II, NRPS, ANSA, and OxyB genes respectively. In this study, it was also found that ANSA gene was more active in the rare actinobacteria, while PKSII genes were frequent in *Streptomyces* sp. However, the NRPS gene was found at almost same frequency in *Streptomyces* and non-*Streptomyces* spp. Approximatley, 46.3% of the isolates were found to be positive for all the four gene clusteres together (Qiu et al., 2015). This implied that gene clusteres are not species specific. In another study of 81 endophytic actinobacteria isolated from *Rhynchotoechum ellipticum* showing antimicrobial and antioxidant activities, presence of gene clusures were analyzed. The results showed that PKS I, PKS II, and NRPS gene clusteres occurred in 19, 51, and 30% of isolates respectively. However, there were 17 isolates possessing all the three genes clusteres. This study also reported that these isolates synthesized several antibiotics: erythromycin, ketoconazole, fluconazole, chloramphenicol, rifampicin and miconazole, phenolic compounds; catechin, kaempferol, chebulagic acid, chlorogenic acid, asiatic acid, ferulic acid, arjunic acid, gallic acid and boswellic acid, and paclitaxel. It is thus evident that these gene clusteres were reposnsible for the production of secondary metabolites (Passari et al., 2018). Frequencies for the presence of biosynthetic genes in the endophytic actinobacteria of the plant *Dracaena cochinchinensis* Lour was 29.4, 70.6, and 23.5%, for PKS-I, PKS-II, and NRPS respectively (Salam et al., 2017). Gene cluster encoding hybrid trans-acyltransferase (AT) polyketide was identified during the genome analysis of endophytic actinobacterium, *Actinoplanes* isolated from the plant *Amphipterygium adstringens* for the first time (Centeno-Leija et al., 2016). In genome analysis of the endophyte, *Streptomyces kebangsaanensis*, it was established that 24 gene clusters were involved in the production of a novel phenanzine. It was also predicted to be involved in the biosynthesis of polyketide, nonribosomal peptide, terpene, bacteriocin, and siderophore (Remali et al., 2017). Presence of PKS and NPRS genes were also repoted in the endophytic actinobacteria of aquatic ecosystem. In a study on endophytic actinobacteria isolated from sea grass *Thalassiahemprichii*, PKS-II, and NRPS genes were detected in almost all of the isolates while PKS-I could be amplified in only half of them (Wu et al., 2012). In another study involving 23 endophytic actinobacteria from tropical plants of Papua New Guinea and Mborokua Island, Solomon Islands, presence of all the three gene clusters PKS I, PKS II and NPRS were reported but no *in vitro* activity was detected. This observation led the authors to interpret that there was no correlation between the presence of gene cluster and the activity (Janso and Carter, 2010). But the inability to detect the bioactive metabolite might be due to non-expression of the genes under the experimental conditions for production.

Available whole genome draft of endophytic actinobacteria also revealed the presence of PKS and NPRS genes suggesting that these microbes are the possible source for many novel bioactive compounds. Genome Sequence of *Microbispora* sp. GMKU 363 harbor three type I PKS gene clusters associated with the synthesis of linfuranone (Komaki et al., 2015). *Streptomyces scabrisporus* NF3, an endophyte isolated from *Amphipterygium adstringens*, carries at least 50 gene clusters for the synthasis of polyketides and nonribosomal peptides (Vazquez-Hernandez et al., 2017). Genome sequence of *Streptomyces* sp. PRh5, a novel endophytic actinobacterium isolated from wild rice root, has been predicted to have putative nigericin and nocardamine biosynthetic gene clusters (Yang et al., 2014). Similarly, *Streptomyces* sp. XY006, an endophyte isolated from Tea (*Camellia sinensis*) had 27 possible secondary metabolite biosynthetic gene clusters encoding for antimycin, melanin, informatipeptin, albaflavenone, and roseoflavin (Vazquez-Hernandez et al., 2017). Genome mining of *Streptomyces wadayamensis* A23, an endophytic strain showed the presence of biosynthetic gene clusteres for antimycins and candicidin (Angolini et al., 2016).

It has been reported that these gene clusters are involved not only in the biosynthesis of bioactive secondary metabolites, but also in the production plant growth promotion factors. Endophytic actinobacteria isolated from *Artemisia annua* possessed herbicidal activity against *Echinochloa crusgalli* and carried PKSI, PKSII, and NRPS gene clusters (Li et al., 2012). *Kibdelosporangium phytohabitans* KLBMP 1111^T^, an endophyte of *Jatropha curcas*, possessed the gene clusters responsible for polyketide and nonribosomal peptide synthesis and also the genes related to the plant growth promoting factors, such as zeatin, 1-aminocyclopropane-1-carboxylate deaminase (ACCD) and siderophore (Qin et al., 2015).

*Streptomyces* sp. XY006 genome sequence revealed the presence of genes associated with synthesis of indole-3-acetic acid, 1-aminocyclopropane-1-carboxylate deaminase, mineral phosphate solubilization, transport and assimilation, and also genes involved in fungal cell-wall degradation, such as a family 19 chitinases (Vazquez-Hernandez et al., 2017). Genome sequence of *Streptomyces* sp. GKU 895 isolated from sugarcane root, showed gene cluster related to plant growth-promoting activity (Kruasuwan et al., 2017). Endophytic actinobacteria isolates of halophytic plant *Salsola imbricate* were found to be active against phytopathogens *Phytophthora capsici* and *Pythium ultimum* and also possessed the biosynthetic gene cluster PKS I, II and NRPSs (Bibi et al., 2018). The screening of the gene clusters in the endophytic actinobacteria can be used as a high throughput method to carry out screening for the presence of bioactive secondary metabolites.

## Opportunities for application of endophytic actinobacteria in agriculture

Use of chemical fertilizers, insecticides, pesticides, and other synthetic agents as nutrients or as biocontrol agents has led to increase in crop productivity and yield (Aktar et al., 2009). However, prolonged use of these substances had adverserly affected the physico-chemical properties of soil, leading to decline in soil fertility and microbial diversity. Also, use of synthetic insecticides and pesticides have resulted in resistant pests and insects along with bioaccumulation of harmful agricultural chemicals and their residues in the consumers invoking serious health concerns (Prashar and Shah, 2016). Evidently, such agricultural practices have come to be considered as non-viable and called for the development and implementation of sustainable alternatives.

Advances in the field of soil microbiome, plant microbiome and increasing understanding of plant-microbe interactions have opened up new vistas for sustainability in agriculture. In this section, we briefly discuss the potential applications of endophytic actinobacteria in protection of plants from diseases, plant nutrition, growth, and tolerence to biotic and abiotic stresses.

### Endophytes in protection against plant diseases

It has been earlier reported in several studies that endophytic actinobacteria can effectively inhibit plant pathogens, thus protect the host-plants from diseases (Taechowisan et al., 2003; Cao et al., 2004). An endophytic actinobacterial strain CEN26 of *Centella asiatica* was inhibitory against the fungal pathogen *Alternaria brassicicola*. It acts by inhibiting germination of conidia and morphological development (Phuakjaiphaeo and Kunasakdakul, 2015). In a study involving 260 isolates of endophytic actinobacteria, majority of them were seen to be inhibiting plant pathogens, and thus had a protective role for protection of the hosts against diseases (Palaniyandi et al., 2013). The type of interaction among the actinobacteria and their host-plants seems to be important in suppression of diseases (Hasegawa et al., 2006; Maggini et al., 2017). Leaf blight disease of rice was cured to a greater extent by *S. platensis* (Wan et al., 2008). Mycolytic, parasitic, and antibiotic activities against *Fusarium* sp. was reported in *Nocardiopsis* sp. (Sabaou et al., 1983). Fifty-five percent of the total endophytes recovered from leaves of *Paeonia lactiflora* and *Trifalium repens* were found to inhibit growth of the phytopathogen, *Rhizoctonia solani* (Gu et al., 2006). Fistupyrone, isolated from *Streptomyces* sp. TP-A0569 inhibited *in vivo* infection of the seedlings of Chinese cabbage by necrotrophic plant pathogen, *Alternaria brassicicola* TP-F0423 (Igarashi et al., 2000). In a recent study, *Streptomyces* sp. PRY2RB2, an endophyte of the medicinal plant *Pseudowintera colorata* (Horopito), a native of New Zealand, have shown inhibitory activity against various phytopathogens: *Neofusicoccum luteum* ICMP 16678, *N. parvum* MM562, *Ilyonectria liriodendri* WPA1C and *Neonectria ditissima* ICMP 14417 (Purushotham et al., 2018). Endophytic actinobacterium, strain AR3, isolated from *Emblica officinalis*, inhibited the growth of *Fusarium oxysporum* (Kamboj et al., 2017). Another endophyte, *Streptomyces* sp. EN27, has also been reported to display antifungal activity against *F. oxysporum* (Conn et al., 2008). He also concluded that resistance to *F. oxysporum* by this strain occurred via an NPR-dependent pathway alongwith salicylic acid but was independent of jasmonic acid/ethylene (JA/ET) pathway. Some other reports of plant protection against phytopathogens have been documented in Table 4.

**Table 4:** Endophytic actinobacteria in protection of host-plants against phytopathogens.

**Endophyte**	**Host-plant**	**Target pathogen**	**References**
*Streptomyces* sp. S30, *Streptomyces* sp. R18(6)	*Lycopersicon esculentum*	*Rhizoctania solani*	Cao et al., 2004; De Oliveira et al., 2010
*Actinoplanes campanulatus, Micromonospora chalcea* and *Streptomyces spiralis*	*Cucumis* sp.	*Pythium aphanidermatum*	El-Tarabily et al., 2010
*Microbispora* sp. A004 and A011, *Streptomyces* sp. A018	*Brassica rapa*	*Plasmodiophora brassicae*	Lee et al., 2008
*Streptomyces diastaticus, Streptomyces fradiae, Streptomyces olivochromogenes, Streptomyces collinus, Streptomyces ossamyceticus Streptomyces griseus* (CC1, CC4, CC12, CC20, CC23, CC29, CC31, CC38, CC41, CC42, CC52 and CC53)	Medicinal plants	*Sclerotium rolfsii, Rhizoktonia solani, Fusarium oxysporum, Alternaria solani*	Singh and Gaur, 2016
*Streptomyces* sp., LBR02, AB131-1 and AB131-2	Host plant information not available	*Xanthomonas oryzae* pv. *oryzae* (Xoo)	Hastuti et al., 2012
*Streptomyces* sp. DBT204	*Solanum lycopersicum*	*Fusarium proliferatum*	Passari et al., 2016
*Leifsonia xyli* BPSAC24,*Streptomyces* sp. BPSAC34	*Eupatorium odoratum, Musa superb, Mirabilis jalapa, Curcuma longa, Clerodendrum colebrookianum, Alstonia scholaris, Centella asiatica,*	*Rhizoctonia solani, Fusarium graminearum, Fusarium oxysporum ciceri, Fusarium prolifratum, Fusarium oxysporum, Fusarium graminearum, Colletotrichum capsici*	Passari et al., 2015a
*Microbispora* sp. isolate 1and 5	*Solanum tuberosum* L.	*Streptomyces scabies*	Goodman, 2014
*Streptomyces* sp. R-5	*Rhododendron* sp.	*Phytophthora cinnamom, Pestalotiopsis sydowiana*	Shimizu et al., 2000
*Streptomyces* sp., MBPu-75 *Streptomyces* sp. MBCu-56	*Cucumis* sp.and *Cucurbita* sp.	*Colletotrichum orbiculare*	Shimizu et al., 2009
*Streptomyces* sp. RM 365	Leguminous plants	*Xanthomonas campestris* pv. *glycine*	Mingma et al., 2014
*Streptomyces thermocarboxydus* TF23, *Streptomyces* sp. TF2, TF21 and TF30	*Lycospersicum esculentum*	*Rhizoctonia solani*	Inderiati and Franco, 2008
*Nocardiopsis* sp. ac9, *Streptomyces* *Violaceorubidus* 6ca11, *Streptomyces* sp. ac19	*Elaeis guineensis* Jacq.	*Ganoderma boninense*	Ting et al., 2014
*Streptomyces mutabilis* CA- 2, *Streptomyces cyaneofuscatus* AA-2	*Aristida pungens* *Cleome arabica* *Solanum nigrum* *Panicum turgidum, Astragallus armatus Peganum harmala* *Hammada scoparia* *Euphorbia helioscopia*	*Rhizoctonia solani*	Goudjal et al., 2014
*Streptomyces felleus* YJ1	*Brassica napus*	*Sclerotinia sclerotiorum*	Cheng et al., 2014
*Streptomyces* sp.	*Garuga pinnata* *Gmelina arborea* *Stephania venosa* *Melastoma malabathricum* *Merremia vitifolia*	*Pectobacterium carotovorum*	Chankhamhaengdecha et al., 2013
*S. griseorubiginosus-*like strain	*Musa acuminata*	*Fusarium oxysporum*	Cao et al., 2005
*Actinoplanes missouriensis* CPWT	*Lupinus termis*	*Plectosporium tabacinum*	El-Tarabily et al., 2010
*Streptomyces* sp. EACK and *Streptomyces* sp. EAOP	*Streptomyces lycopersicum* Mill.	*Ralstonia solanacearum*	Sreeja and Gopal, 2013
*Streptomyces* sp. AzR-051, AzR- 049, AzR- 010	*Azadirachta indica* A. Juss.	*Alternaria alternata*	Verma et al., 2011
*Streptomyces* sp.AOK-30	*Kalmia latifolia* L.	*Pestalotiopsis sydowiana*	Nishimura et al., 2002
*Streptomyces violaceusniger*	Trees of Dehradun	*Phanerochaete chrysosporium, Coriolus versicolor, Gloephyllum trabeum*	Shekhar et al., 2006
*Streptomyces* sp. PRY2RB2	*Pseudowintera colorata*	*Neofusicoccum luteum* ICMP 16678, *Nocardia parvum* MM562, *Ilyonectria liriodendri* WPA1C, *Neonectria ditissima* ICMP 14417	Purushotham et al., 2018

### Endophytic actinobacteria in plant growth promotion

Endophytic actinobacteria are known for their potential to affect plant growth and nutrient uptake (Rajkumar et al., 2006). They are reported as plant growth promoters in cereals and legumes (Mishra et al., 1987; Salam et al., 2016). In some studies, it was observed that endophytes are very good producers of plant growth promoting factors like phytohormones. Indole acetic acid (IAA) is among the highly reported growth regulator from endophytic actinobacteria (Khamna et al., 2010; Palaniyandi et al., 2013). Endophytic *Streptomyces* strains producing IAA significantly improved the growth of tomato plants (Verma et al., 2011). There are reports suggesting that the endophytic actinobacteria isolated from one plant could promote growth of the other plants. For instance, rhizospheric endophytic actinobacteria of Yam plant of Yeoju, South Korea, enhanced the growth of *Arabidopsis thaliana* by producing IAA, phosphate solubilization, and prominent 1-aminocyclopropane-1-carboxylic acid (ACC) deaminase activity (Palaniyandi et al., 2013). However, some of the studies suggested that there is negative impact of IAA as it has been verified as one of the virulence factors in some phytpathogenic actinobacteria (Vandeputte et al., 2005). Endophytic actinobacteria of wheat crop, for example, *S. olivaceoviridis, S. rimosus* and *S. rochei* produced auxins, gibberellins, and cytokinin-like substances that positively influenced plant growth (Aldesuquy et al., 1998). It has been suggested that formulations of endophytic actinobacteria can be a potential step to promote sustainable agriculture (Hasegawa et al., 2006; Palaniyandi et al., 2013). Plant growth promoters, Pteridic acids A and B, isolated from endophytic *S. hygroscopicus* TP-A045 had auxin-like activity and helped in the improved formation of adventitious root in hypocotyls of kidney beans. They showed potency comparable to the auxins (Igarashi et al., 2002). Another *Streptomyces* sp. MBR-52 also augmented the emergence and elongation of plant adventitious roots (Hasegawa et al., 2006; Meguro et al., 2006). Ethylene a phytohormone is responsible for the physiological responses to both abiotic and biotic environmental stresses to plants (Sun et al., 2006). Extreme physiological conditions like temperature, drought and salinity can induce the production of ethylene. Endophytic actinobacteria residing in such plants might lessen the negative impact of stress by production of enzyme ACC deaminase. This enzyme hydrolyzes 1-aminocyclopropane-1-carboxylic acid (ACC) and hence decreases the ethylene production in plants. In some studies, it is also reported that this enzyme is capable of hydrolyzing ACC into a-ketobutyrate and ammonia, and provide nitrogen to the microorganisms for their growth (Viterbo et al., 2010; Xing et al., 2012).

Phosphorus is one of the essential elements for plants necessary for various biological processes like transport of glucose, stimulation of cell proliferation and promotion of organ development (Ahemad and Kibret, 2014). But the phosphorus available in the soil is not accessible to plants directly (Ezawa et al., 2002). Endophytic actinobacteria play important role in solubilization of phosphates and enhance its availability to plants by means of acidification, chelation, redox changes and mineralization of organic phosphorus (Nautiyal et al., 2000; Van der Hiejden et al., 2008). Phosphate solubilization activity along with the phytase secretion was reported from endophytic *Streptomyces* sp. isolated from *Triticum aestivum* plant. It has shown significant plant growth improvement (Jog et al., 2014).

Besides phosphorus, iron also plays an imperative role in the physiological processes and enzymatic activities in plants (Bothwell, 1995). For iron to be taken up by plants, it needs to be solubilised. The availability of iron to plant roots was enhanced by siderophores produced by microorganisms in the form of bacterial siderophore-iron complex, or phytosiderophore-iron complex (Rajkumar et al., 2009; Ma et al., 2011). *S. acidiscabies* E13 being an excellent producer of siderophore, promotes the growth of *Vigna unguiculata* under nickel stress conditions (Dimkpa et al., 2009; Sessitsch et al., 2013). In a recent study on the community of endophytic actinobacteria of New Zealand native medicinal plant *Pseudowintera colorata* (Horopito), *Streptomyces* sp. UKCW/B and *Nocardia* sp. TP1BA1B were found to solubilise phosphate and produce siderophores (Purushotham et al., 2018).

Some reports had shown direct benefits of endophyte on plants by tissue culture. For example, inoculation of halophytic plants with plant growth-promoting (PGP) actinobacteria was reported for the improvement of salt tolerance in plants (Qin et al., 2017). *Streptomyces coelicolor* (MAR1), an endophyte isolated from mangrove plants of Pichavaram, Tamilnadu (India) was reported to be active against various bacterial and fungal pathogens. It was also tolerant for high salt concentration and high pH (Gayathri and Muralikrishnan, 2013).

The endophytic actinobacteria also possess the ability to produce growth inhibitors in order to improve health of the host-plants. Some herbicidal compounds had been reported from the endophytic actinobacteria. Herbicidin H isolated from *Streptomyces* sp. SANK 63997, an endophyte of *Setaria viridis* var. *pachystachys*, gamma-glutamylmethionine sulfoximine from *Microbispora* sp. residing in *Carex kobomugi* are some of the examples of herbicides of endophytic origin (Okazaki, 2003). A plant growth inhibitor was isolated from actinobacteria *Dactylosporangium* sp. of *Cucubalus* sp. found to inhibit germination of *Brassica rapa* (Furumai et al., 2003). Clethramycin, a pollen tube growth inhibitor extracted from the fermentation broth of *S. hygroscopicus*, was isolated from the roots of *Clethra barbinervis*. This metabolite has mode of action similar to inhibition of MAP kinase by staurosporine and herbimycin (Igarashi et al., 2003). Endophenazines A-D, produced by *S. anulatus* possessed herbicidal activity against *Lemna minor* (Gebhardt et al., 2002).

Besides these beneficial properties, a few phytotoxins have also been reported from the endophytic actinobacteria (Berdy, 2012), but majorly, they play beneficial roles. Thus, it can be safely summed up that there is enormous potential for application of endophytic actinobacteria in agriculture. Some more studies of plant growth promotion by endophytic actinobacteria are reported in Table 5.

**Table 5:** Plant growth promoting activities of endophytic actinobacteria.

**Plant growth promotion**			
**Plant growth promoting factor**	**Endphytic actinobacteria**	**Plant source**	**References**
Nitrogen Fixation	Actinomycetes strains Dll, G2	*Casuarina equisetifolia*	Gauthier et al., 1981
IAA and siderophore	*Streptomyces* sp. CMU-PA101 and *Streptomyces* sp. CMU-SK126	*Curcuma mangga*	Khamna et al., 2009
Solubilization of phosphate, production of phytase, chitinase, IAA, siderophore and malate	*Streptomyces* sp. mhcr0816, mhce0811	*Triticum aestivum*	Jog et al., 2014
Production of IAA and ACC deaminase	*Actinoplanes campanulatus, Micromonospora chalcea, Streptomyces spiralis*	*Cucumis sativus*	El-Tarabily et al., 2010
Production of IAA	*Streptomyces* sp. PT2	Plants of Algerian Sahara	Goudjal et al., 2013
Production of chitinase, phosphatase and siderophore	*Streptomyces* sp. AB131-1, LBR02	Isolates of Microbiology Laboratory, Bogor Agricultural University	Hastuti et al., 2012
Production of rooting-promoting plant hormones	*Streptomyces* sp. MBR-52	*Rhododendron ferrugineum*	Meguro et al., 2006
Solubilization of phosphate, production of siderophores, HCN, ammonia, production of chitinase and IAA	*Streptomyces* sp. BPSAC34, *Leifsonia xyli* BPSAC24, *Microbacterium* sp. BPSAC 21, 27, 28 and 29	Medicinal plants	Passari et al., 2015b
IAA	*Streptomyces* sp.*, Nocardia* sp., *Nocardiopsis* sp., *Spirillospora* sp., *Microbispora* sp. *and Micromonospora* sp.	*Citrus reticulata*	Shutsrirung et al., 2013
Siderophores	*Streptomyces* sp. GMKU 3100	*Oryza sativa* L. cv. KDML105	Rungin et al., 2012
Indole-3-acetic acid (IAA), hydroxamate and catechol type siderophore, protease	*Streptomyces* sp. S4202, *Nonomuraea* sp. S3304, *Actinomadura* sp. S4215*, Pseudonocardia* sp. S4201	*Aquilaria crassna*	Nimnoi et al., 2010
Glucoamylase	*Streptosporangium* sp. L21	*Zea mays*	De Araujo et al., 2000; Stamford et al., 2002
Cell wall degrading enzymes	*Streptomyces galbus* R-5	Rhododendron seedlings	Minamiyama et al., 2003
Endo- chitinase enzymes	*Streptomyces violaceusniger* XL- 2	Trees of Dehradun	Shekhar et al., 2006
IAA, siderophore production, Phosphate solubilisation	*Streptomyces sp*. R18(6)	*Lycopersicon esculentum*	De Oliveira et al., 2010
Homoserine lactone degrading enzymes	*Streptomyces* sp. LPC026, LPC029, PC005, PC052, and PC053	*Garuga pinnata, Gmelina arborea, Stephania venosa, Melastoma malabathricum, Merremia vitifolia*	Chankhamhaengdecha et al., 2013
Herbicidin H (herbicide)	*Streptomyces* sp. strain SANK 63997	*Setaria viridis* var*. pachystachys*	Okazaki, 2003
Cellulase, Xylanase, Lignolytic activity	*Nocardiopsis* sp. ac9, *Streptomyces, Violaceorubidus* 6ca11, *Streptomyces* sp. ac19	*Elaeis guineensis* Jacq.	Ting et al., 2014
Phosphate solubilisation and seiderophore production	*Streptomyces* sp. UKCW/B, *Nocardia* sp. TP1BA1B	*Pseudowintera colorata*	Purushotham et al., 2018

## Environmental applications of endophytic actinobacteria

Some endophytic actinobacteria isolated from the plant, *Salix caprea*, were reported to release metal binding secondary metabolites that mobilize zinc (Zn) and cadmium (Cd) from the soil, enhancing their accumulation in the leaves of this plant (Kuffner et al., 2010). In another study involving the endophyte *S. tendae* from sunflower plant, it was reported to mediate phytoremediation of Cd (Dimkpa et al., 2009). These studies have clearly established the potential of endophytic actinobacteria in removal of heavy metals from the soil and their increased uptake by plants growing in the metal-contaminated soil. A combinatorial study of plants and their endophytes can be explored for their application in remediation of heavy metals from contaminated soil sites and consequent improvement in soil health.

A few reports on the potential application of endophytic actinobacteria in pesticide bioremediation have also appeared. Chlordane, pesticide banned due to its adverse effect on human health and environment, was successfully removed by using a mixed culture of actinobacteria. It was shown that the mixed culture produced bioemulsifiers that facilitated bioavailability and uptake of the pesticide (Fuentes et al., 2017).

Endophytic *Streptomyces* sp. had been reported in improving phytoremediation efficiency of petroleum contaminated soil by their petroleum degradation ability (Baoune et al., 2017). Significant biosurfactant and biodegradation activities had been reported from the endophyte *Nocardia* sp. A9. Furthermore, it was able to degrade plastic and petroleum by 22 and 10% on weight loss basis respectively (Singh and Sedhuraman, 2015).

## Conclusions and future perspectives

Microbes and plants are keys to sustenance of life on the planet earth. They are the drivers of natural processes, like biogeochemical cycles, maintenance of various ecological habitats (supporting specific flora and fauna under such special niches), production of oxygen, utilization of carbon dioxide, production of organic compounds used as food, feed, and medicine. These naturally thriving and beautifully maintained biosystems have come under serious threat due to deleterious consequences of rampant industrialization and unthoughtful use of myriad of chemicals for human, animal, and agriculture purposes. These problems have drawn attention of the researchers to find appropriate solutions. It is during such endeavors that we are learning more about various biotic and abiotic factors, which interact in a very rationale and scientific manner to balance and sustain each other. Endophytic actinobacteria and their host plants provide an exciting model to explore and to understand their biology and chemistry to develop suitable, non-deleterious applications for human health, agriculture and environment.

While the genus *Streptomyces* had been reported to be the most abundant, the non-*Streptomyces* category was also frequently reported in the endophyte of various categories of plants from extreme habitats (Table 1). The biosynthetic gene clusters (BGCs) of these isolates are largely unexplored. But significant diversity in chemical classes was evident from the reports summarized in Tables 2, 3. The therapeutic properties of these compounds strongly suggested abudance of potentially novel drug molecules in the expansive chemical repertoire of endophytic actinobacteria. The data presented in Tables 4, 5 demonstrated important roles of endophytic actinobacteria in plant protection and growth, thereby their significance in agriculture.

From the studies analyzed in this review, it is evident that the plants from the extreme environments are potential source for novel and widely diverse endophytic actinobacteria. While these natural reservoirs remain largely untapped as of now, they seem to be drawing considerable attention of the scientific community in present times. Furthermore, progress in endophytic actinobacterial diversity will depend on the advancements in tools and technology for both culturable and non-culturable approaches. This would enhance the repertoire of culturable endophytic actinobacteria from the special habitats besides expanding the information on non-culturable forms. Advancement in the genomic science and technology can be expected to offer rapid means to establish the endophytic actinobacterial diversity in extreme habitats and to indentify potentially useful strains based on the detection of BGCs or other genetic determinants.

Due to multiple drug resistant (MDR) and extensive drug resistant (XDR) pathogens on the scene and the problems associated with the treatment and management of the diseases caused by “ESKAPE” pathogens (*Enterococcus faecium, Staphylococcus aureus, Klebsiella pnumoniae, Acinetobacter baumannii, Pseudomonas aeruginosa, and Enterobacter* species), the need for discovery and development of novel drugs is more urgent than ever before. The diverse and novel endophytic actinobacteria from the special habitats offer unparallel opportunities for mining drugs and therapeutics. Besides application in medicine, the novel bioactive molecules may be useful for agriculture purposes for their ability to promote plant growth; ward off phytopathogens, insects and pests; enhance nutrient uptake and help maintain crop productivity under severe abiotic stress of salinity, draft and water logging. Also, these endophytic actinobacteria may play crucial roles in maintenance and sustenance of specific natural habitats but these endeavors call upon extensive scientific investigation to develop the necessary knowledge base.

It can thus be summed up that the novelty and the diversity of the endophytic actinobacterial strains and their bioactive molecules offer tremendous opportunities to address the current and future needs in medicine, agriculture, and environment.

## Author contributions

RS: collection of literature, preparation of the initial draft, support during course of preparation of the article. AD: structure and content of the review, correction of the initial draft, writing, and revisiion of the final version.

### Conflict of interest statement

The authors declare that the research was conducted in the absence of any commercial or financial relationships that could be construed as a potential conflict of interest.

## Footnotes

^1^Available online at: http://www.chemicalbook.com/ChemicalProductProperty_EN_CB6965824.htm

^2^Available online at: http://www.chemspider.com/Chemical-Structure.4450703.html

^3^Available online at: https://www.molinstincts.com/chemical-structure/7-Methoxy-3-6-3-4-tetrahydroxyflavone-cstr-CT1105232405.html

## References

[B1] Abreu-Tarazi M. F. Navarrete A. A. Andreote F. D. Almeida C. V. Tsai S. M. Almeida M. (2010). Endophytic bacteria in long-term *in vitro* cultivated axenic pineapple microplants revealed by PCR DGGE. World, J. Microbiol. Biotechnol. 26, 555–560. 10.1007/s11274-009-0191-3

[B2] Ahemad M. Kibret M. (2014). Mechanisms and applications of plant growth promoting rhizobacteria: current perspective. J. King Saud Univ. Sci. 26, 1–20. 10.1016/j.jksus.2013.05.001

[B3] Ahmad T. Singh S. B. Pandey S. (2013). Phytochemical screening and physicochemical parameters of crude drugs: a brief review. Int. J. Pharm. Res. Rev. 2, 53–60.

[B4] Akshatha V. J. Nalini M. S. D'Souza C. Prakash H. S. (2014). *Streptomycete* endophytes from anti-diabetic medicinal plants of the Western Ghats inhibit alpha-amylase and promote glucose uptake. Lett. Appl. Microbiol. 58, 433–439. 10.1111/lam.12209 24330131

[B5] Aktar M. W. Sengupta D. Chowdhury A. (2009). Impact of pesticides use in agriculture: their benefits and hazards. Interdiscip. Toxicol. 2, 1–12. 10.2478/v10102-009-0001-7 21217838 PMC2984095

[B6] Aldesuquy H. S. Mansour F. A. AboHamed S. A. (1998). Effect of the culture filtrates of *Streptomyces* on growth and productivity of wheat plants. Folia Microbiol. 43, 465–470. 10.1007/BF02820792

[B7] Alexander D. E. Fairbridge R. W. (1999). Encyclopedia of Environmental Science. Dordrecht: Springer.

[B8] Alongi D. M. (1988). Bacterial productivity and microbial biomass in tropical mangrove sediments. Microb. Ecol. 15, 59–79. 10.1007/BF02012952 24202863

[B9] Alongi D. M. (2002). Present state and future of the world's mangrove forests. Environ. Conserv. 29, 331–349. 10.1017/S0376892902000231

[B10] Alshaibani M. Zin N. Jalil J. Sidik N. Ahmad S. J. Kamal N. (2017). Isolation, purification, and characterization of five active diketopiperazine derivatives from endophytic *Streptomyces* SUK 25 with antimicrobial and cytotoxic activities. J. Microbiol. Biotechnol. 27, 1249–1256. 10.4014/jmb 28535606

[B11] Amar M. B. Elleuch L. Abd-Alla H. I. Najah S. Chakchouk A. Damak M. (2012). The new *Streptomyces* sp. TN605 strain secretes simultaneously three active compounds and a high level of the interesting pharmaceutical industry intermediate: 2-Hydroxyphenylacetic Acid. Bull. Environ. Pharmacol. Life Sci. 1, 48–56.

[B12] Amrita K. Nitin J. Devi C. S. (2012). Novel bioactive compounds from mangrove derived actinomycetes. Int. Res. J. Pharm. 3, 25–29.

[B13] Andreote F. D. Gumiere T. Durrer A. (2014). Exploring interactions of plant microbiomes. Sci. Agric. 71, 528–539. 10.1590/0103-9016-2014-0195

[B14] Angolini C. F. F. Gonçalves A. B. Sigrist R. Paulo B. S. Samborskyy M. Cruz P. L. R. (2016). Genome mining of endophytic *Streptomyces wadayamensis* reveals high antibiotic production capability. J. Braz. Chem. Soc. 27, 1465–1475. 10.5935/0103-5053.20160180

[B15] Anisimova M. Gascuel O. (2006). Approximate likelihood ratio test for branchs: a fast, accurate and powerful alternative. Syst. Biol. 55, 539–552. 10.1080/10635150600755453 16785212

[B16] Ara I. Kudo T. Matsumoto A. Takahashi Y. Omura S. (2007). *Nonomuraea maheshkhaliensis* sp. nov., a novel actinomycete isolated from mangrove rhizosphere mud. J. Gen. Appl. Microbiol. 53, 159–166. 10.2323/jgam.53.159 17726296

[B17] Asaf S. Khan M. A. Khan A. L. Waqas M. Shahzad R. Kim A. Y. (2017). Bacterial endophytes from arid land plants regulate endogenous hormone content and promote growth in crop plants: an example of *Sphingomonas* sp. *and Serratia marcescens*. J. Plant Interact. 12, 31–38. 10.1080/17429145.2016.1274060

[B18] Ayuso-Sacido A. Genilloud O. (2005). New PCR primers for the screening of NRPS and PKS-I systems in actinomycetes: detection and distribution of these biosynthetic gene sequences in major taxonomic groups. Microb. Ecol. 49, 10–24. 10.1007/s00248-004-0249-6 15614464

[B19] Azman A. S. Othman I. Velu S. S. Chan K. G. Lee L. H. (2015). Mangrove rare actinobacteria: taxonomy, natural compound, and discovery of bioactivity. Front. Microbiol. 6:856. 10.3389/fmicb.2015.00856 26347734 PMC4542535

[B20] Babu K. V. P. Chandrakala C. Ram T. Latha P. C. (2014). Plant growth promoting, biocontrol and antioxidant activities of endophytic actinomycetes of rice. Agrotechnol. 2:239 10.4172/2168-9881.S1.008

[B21] Baltz R. H. (2008). Renaissance in antibacterial discovery from actinomycetes. Curr. Opin. Pharmacol. 8, 557–563. 10.1016/j.coph.2008.04.008 18524678

[B22] Baoune H. Ould El Hadj-Khelil A. Pucci G. Sineli P. Loucif L. Polti M. A. (2017). Petroleum degradation by endophytic *Streptomyces* sp. isolated from plants grown in contaminated soil of southern Algeria. Ecotoxicol. Environ. Saf. 147, 602–609. 10.1016/j.ecoenv.2017.09.013 28923725

[B23] Barka E. A. Vatsa P. Sanchez L. Gaveau-Vaillant N. Jacquard C. Meier-Kolthoff J. P. (2016). Taxonomy, physiology, and natural products of actinobacteria. Microbiol. Mol. Biol. Rev. 25, 80, 1–43. 10.1128/MMBR.00019-15 26609051 PMC4711186

[B24] Baskaran R. Mohan P. M. Sivakumar K. Raghavan P. Sachithanandam V. (2012). Phyllosphere microbial populations of ten true mangrove species of the Andaman Island. Int. J. Microbiol. Res. 3, 124–127. 10.5829/idosi.ijmr.2012.3.2.6221

[B25] Berdy J. (2012). Thoughts and facts about antibiotics: where we are now and where we are heading. J. Antibiot. 65, 385–395. 10.1038/ja.2012.27 22511224

[B26] Bernstein L. Bosch P. Canziani O. Chen Z. Christ R. Davidson O. (2007). IPCC: Climate Change: Synthesis Report. An Assessment Report of the Intergovernmental Panel on Climate Change. Geneva: Intergovernmental Panel on Climate Change Available online at: https://www.ipcc.ch/pdf/assessment-report/ar4/syr/ar4_syr_full_report.pdf

[B27] Bian G. K. Feng Z. Z. Qin S. Xing K. Wang Z. Cao C. L.. (2012a). *Kineococcus endophytica* sp. nov., a novel endophytic actinomycete isolated from a coastal halophyte in Jiangsu. Antonie van Leeuwenhoek 102, 621–628. 10.1007/s10482-012-9757-4 22669199

[B28] Bian G. K. Qin S. Yuan B. Zhang Y. J. Xing K. Ju X. J.. (2012b). *Streptomyces phytohabitans* sp. nov., a novel endophytic actinomycete isolated from medicinal plant Curcuma phaeocaulis. Antonie van Leeuwenhoek 102, 289–296. 10.1007/s10482-012-9737-8 22527624

[B29] Bibi F. Strobel G. A. Naseer M. I. Yasir M. Khalaf Al-Ghamdi A. A. Azhar E. I. (2018). Microbial flora associated with the halophyte- *Salsola imbricate* and its biotechnical potential. Front. Microbiol. 9:65. 10.3389/fmicb.2018.00065 29445362 PMC5797760

[B30] Bieber B. Nuske J. Ritzau M. Grafe U. (1998). Alnumycin, a new naphthoquinone antibiotic produced by an endophytic *Streptomyces* sp. J. Antibiot. 51, 381–82. 9589078 10.7164/antibiotics.51.381

[B31] Boonsnongcheep P. Nakashima T. Takahashi Y. Prathanturarug S. (2017). Diversity of endophytic actinomycetes isolated from roots and root nodules of *Pueraria candollei* Grah. ex Benth. and the analyses of their secondary metabolites. Chiang Mai. J. Sci. 44, 1–14.

[B32] Bothwell T. H. (1995). Overview and mechanisms of iron regulation. Nutr. Rev. 53, 237–245. 8577406 10.1111/j.1753-4887.1995.tb05480.x

[B33] Bulgari D. Casati P. Brusetti L. Quaglino F. Brasca M. Daffonchio D.. (2009). Endophytic bacterial diversity in grapevine (*Vitis vinifera* L.) leaves described by 16S rRNA gene sequence analysis and length heterogeneity-PCR. J Microbiol. 47, 393–401. 10.1007/s12275-009-0082-1 19763412

[B34] Bunyoo C. Duangman K. Nuntagij A. Thamchaipenet A. (2009). Characterisation of endophytic actinomycetes isolated from wattle trees (*Acacia auriculiformis* A. Cunn. ex Benth.) in Thailand. Thai. J. Genet. 2, 155–163. 10.14456/tjg.2009.1

[B35] Cane D. E. Walsh C. T. (1999). The parallel and convergent universes of polyketide synthases and nonribosomal peptide synthetases. Chem. Biol. 6, 319–325. 10631508 10.1016/s1074-5521(00)80001-0

[B36] Cao L. Qiu Z. You J. Tan H. Zhou S. (2004). Isolation and characterization of endophytic *Streptomyces* strains from surface-sterilized tomato (*Lycopersicon esculentum*) roots. Lett. Appl. Microbiol. 39, 425–430. 10.1111/j.1472-765X.2004.01606.x 15482433

[B37] Cao L. Qiu Z. You J. Tan H. Zhou S. (2005). Isolation and characterization of endophytic *Streptomycet*e antagonists of *Fusarium* wilt pathogen from surface-sterilized banana roots. FEMS Microbiol. Lett. 247, 147–152. 10.1016/j.femsle.2005.05.006 15935565

[B38] Cao Y. R. Jiang Y. Jin R. X. Han L. He W. X. Li Y. L. (2012). *Enteractinococcus coprophilus* gen. nov., sp. nov., of the family *Micrococcaceae* isolated from *Panthera tigris* amoyensis feces, transfer of *Yaniella fodinae* Dhanjal et al. 2011 to the genus *Enteractinococcus as* as *Enteractinococcus fodinae* comb. nov. Int. J. Syst. Evol. Microbiol. 62, 2710–2716. 10.1099/ijs.0.034249-0 22228667 PMC4080730

[B39] Caruso M. Colombo A. L. Crespiperellino N. Fedeli L. Malyszko J. Pavesi A. (2000). Studies on a strain of *Kitasatospora* sp. paclitaxel producer. Ann. Microbiol. 50, 89–102.

[B40] Castillo U. F. Strobel G. A. Ford E. J. Hess W. M. Porter H. Jensen J. B.. (2002). Munumbicins, wide-spectrum antibiotics produced by *Streptomyces* NRRL 30562, endophytic on *Kennedia nigriscans*. Microbiology 148, 2675–2785. 10.1099/00221287-148-9-2675 12213914

[B41] Castillo U. F. Strobel G. A. Mullenberg K. Condron M. M. Teplow D. B. Folgiano V. (2006). Munumbicins E-4 and E-5: novel broad-spectrum antibiotics from *Streptomyces* sp. NRRL 3052. FEMS Microbiol. Lett. 25, 296–300. 10.1111/j.1574-6968.2005.00080.x 16448509

[B42] Castillo U. Harper J. K. Strobel G. A. Sears J. Alesi K. Ford E.. (2003). Kakadumycins, novel antibiotics from *Streptomyces* sp NRRL 30566, an endophyte of *Grevillea pteridifolia*. FEMS Microbiol. Lett. 224, 183–190. 10.1016/S0378-1097(03)00426-9 12892881

[B43] Centeno-Leija S. Vinuesa P. Rodríguez-Pena K. Trenado-Uribe M. Cardenas-Conejo Y. Serrano-Posada H.. (2016). Draft genome sequence of an endophytic *Actinoplanes* species, encoding uncommon trans-acyltransferase polyketide synthases. Genome Announc. 4:e00164–e00116. 10.1128/genomeA.00164-16 27013046 PMC4807235

[B44] Chankhamhaengdecha S. Hongvijit S. Srichaisupakit A. Charnchai P. Panbangred W. (2013). Endophytic actinomycetes: a novel source of potential acyl homoserine lactone degrading enzymes. Biomed. Res. Int. 2013:782847. 10.1155/2013/782847 23484156 PMC3581087

[B45] Chen H. H. Qin S. Li J. Zhang Y. Q. Xu L. H. Jiang C. L.. (2009). *Pseudonocardia endophytica* sp. nov., isolated from the pharmaceutical plant Lobelia clavata. Int. J. Syst. Evol. Microbiol. 59, 559–563. 10.1099/ijs.0.64740-0 19244441

[B46] Chen M. H. Zhang L. Zhang X. (2011). Isolation and inoculation of endophytic actinomycetes in root nodules of *Elaeagnus angustifolia*. Mod. Appl. Sci. 5, 264–267. 10.5539/mas.v5n2p264

[B47] Cheng G. Huang Y. Yang H. Liu F. (2014). *Streptomyces felleus* YJ1: potential biocontrol agents against the *Sclerotinia* stem rot (*Sclerotinia sclerotiorum*) of oilseed rape. J. Agric. Sci. 6, 91–98. 10.5539/jas.v6n4p91

[B48] Chevenet F. Brun C. Banuls A. L. Jacq B. Chisten R. (2006). TreeDyn: towards dynamic graphics and annotations for analyses of trees. BMC Bioinf. 7:439. 10.1186/1471-2105-7-439 17032440 PMC1615880

[B49] Chung K. T. Wong T. Y. Wei C. I. Huang Y. W. Lin Y. (1998). Tannins and human health: a review. Crit. Rev. Food Sci. Nutr. 38, 421–464. 10.1080/10408699891274273 9759559

[B50] Conn V. M. Walker A. R. Franco C. M. M. (2008). Endophytic actinobacteria induce defense pathways in *Arabidopsis thaliana*. Mol. Plant Microbe Interact. 21, 208–218. 10.1094/MPMI-21-2-0208 18184065

[B51] Conn V. M. Franco C. M. M. (2004). Analysis of the endophytic actinobacterial population in the roots of wheat (*Triticum aestivum L*.) by terminal restriction fragment length polymorphism and sequencing of 16S rRNA clones. Appl. Environ. Microbiol. 70, 1787–1794. 10.1128/AEM.70.3.1787-1794.2004 15006805 PMC368409

[B52] Coombs J. T. Franco C. M. M. (2003). Isolation and identification of actinobacteria from surface-sterilized wheat roots. Appl. Environ. Microbiol. 69, 5603–5608. 10.1128/AEM.69.9.5603-5608 12957950 PMC194995

[B53] Corre C. Challis G. L. (2007). Heavy tools for genome mining. Chem. Biol. 14, 7–9. 10.1016/j.chembiol.2007.01.001 17254946

[B54] Crosa J. H. Walsh C. T. (2002). Genetics and assembly line enzymology of siderophore biosynthesis in bacteria. Microbiol. Mol. Biol. Rev. 66, 223–249. 10.1128/MMBR.66.2.223-249.2002 12040125 PMC120789

[B55] De Araujo J. M. da Silva A. Azevedo J. L. (2000). Isolation of endophytic actinomycetes from roots and leaves of maize (*Zea mays* L.). Braz. Arch. Biol. Technol. 43:4 10.1590/S1516-89132000000400016

[B56] De Bary A. (1866). Morphologie und Physiologie der Pilze, Flechten und Myxomyceten Vol. 2. Leipzig: Hofmeister's Handbook of Physiological Botany.

[B57] De Oliveira M. F. da Silva G. M. Sand S. T. V. D. (2010). Anti-phytopathogen potential of endophytic actinobacteria isolated from tomato plants (*Lycopersicon sculentum*) in southern Brazil, and characterization of *Streptomyces* sp. R18, a potential biocontrol agent. Res. Microbiol. 161, 565–572. 10.1016/j.resmic.2010.05.008 20542109

[B58] Dereeper A. Audic S. Claverie J. M. Blanc G. (2010). BLAST-EXPLORER helps you building datasets for phylogenetic analysis. BMC Evol. Biol. 10, 1–6. 10.1186/1471-2148-10-8 20067610 PMC2821324

[B59] Dereeper A. Guignon V. Blanc G. Audic S. Buffet S. Chevenet F (2008). Phylogeny.fr: Robust phylogenetic analysis for the non-specialist. Nucleic Acids Res. 1, W465–W469. 10.1093/nar/gkn180 PMC2447785 18424797

[B60] Dimkpa C. O. Merten D. Svatos A. Büchel G. Kothe E. (2009). Siderophores mediate reduced and increased uptake of cadmium by *Streptomyces tendae* F4 and sunflower (*Helianthus annuus*), respectively. J. Appl. Microbiol. 107, 1687–1696. 10.1111/j.1365-2672.2009.04355.x 19457036

[B61] Dinesh R. Srinivasan V. T E. S. Anandaraj M. Srambikkal H. (2017). Endophytic actinobacteria: diversity, secondary metabolism and mechanisms to unsilence biosynthetic gene clusters. Crit. Rev. Microbiol. 43, 546–566. 10.1080/1040841X.2016.1270895 28358596

[B62] Ding L. Münch J. Goerls H. Maier A. Fiebig H. H. Lin W. H.. (2010). Xiamycin, a pentacyclic indolosesquiterpene with selective anti- HIV activity from a bacterial mangrove endophyte. Bioorg. Med. Chem. Lett. 20, 6685–6687. 10.1016/j.bmcl.2010.09.010 20880706

[B63] Dini-Andreote F. Andreote F. D. Araújo W. L. Trevors J. T. van Elsas J. D. (2012). Bacterial genomes: habitat specificity and uncharted organisms. Microb. Ecol. 64, 1–7. 10.1007/s00248-012-0017-y 22395783 PMC3375415

[B64] Doroghazi J. R. Metcalf W. W. (2013). Comparative genomics of actinomycetes with a focus on natural product biosynthetic genes. BMC Genomics 14, 1–13. 10.1186/1471-2164-14-611 24020438 PMC3848822

[B65] Drigo B. Van Veen J. A. Kowalchuk G. A. (2009). Specific rhizosphere bacterial and fungal groups respond to elevated atmospheric CO2. ISME J. 3, 1204–1217. 10.1038/ismej.2009.65 19536195

[B66] Du H. J. Zhang Y. Q. Liu H. Y. Su J. Wei Y. Z. Ma B. P.. (2013). *Allonocardiopsis opalescens* gen. nov., sp. nov., a new member of the suborder Streptosporangineae, from the surface- sterilized fruit of a medicinal plant. Int. J. Syst. Evol. Microbiol. 63, 900–904. 10.1099/ijs.0.041491-0 22634703

[B67] Dudeja S. S. Giri R. (2014). Beneficial properties, colonization, establishment and molecular diversity of endophytic bacteria in legume and non-legume. Afr. J. Microbiol. Res. 8, 1562–1572. 10.5897/AJMR2013.6541

[B68] Eccleston G. P. Brooks P. R. Kurtböke D. I. (2008). The occurrence of bioactive *Micromonosporae* in aquatic habitats of the Sunshine Coast in Australia. Mar. Drugs. 6, 243–261. 10.3390/md20080012 18728727 PMC2525489

[B69] Edgar R. C. (2004). MUSCLE: multiple sequence alignment with high accuracy and high throughput. Nucleic Acids Res. 32, 1792–1797. 10.1093/nar/gkh340 15034147 PMC390337

[B70] El-Gendy M. M. El-Bondkly A. M. (2010). Production and genetic improvement of a novel antimycotic agent, saadamycin, against dermatophytes and other clinical fungi from endophytic *Streptomyces* sp. Hedaya48. J. Ind. Microbiol. Biotechnol. 37, 831–841. 10.1007/s10295-010-0729-2 20458610

[B71] El-Tarabily K. A. (2003). An endophytic chitinase-producing isolate of *Actinoplanes missouriensis*, with potential for biological control of root rot of lupin caused by *Plectosporium tabacinum*. Aust. J. Bot. 51, 257–266. 10.1071/BT02107

[B72] El-Tarabily K. Hardy G. E. J. Sivasithamparam K. (2010). Performance of three endophytic actinomycetes in relation to plant growth promotion and biological control of *Pythium aphanidermatum*, a pathogen of cucumber under commercial field production conditions in the United Arab Emirates. Eur. J. Plant Pathol. 128, 527–539. 10.1007/s10658-010-9689-7

[B73] Ernawati M. Solihin D. D. Lestari Y. (2016). Community structures of endophytic actinobacteria from medicinal plant *Centella asiatica* L. Urban-based on metagenomic approach. Int. J. Pharm. Pharm. Sci. 8, 292–297. Available online at: https://innovareacademics.in/journals/index.php/ijpps/article/view/9568

[B74] Ezawa T. S Smith S. E. Smith F. A. (2002). P metabolism and transport in AM fungi. Plant Soil. 244, 221–230. 10.1023/A:1020258325010

[B75] Ezra D. Castillo U. F. Strobel G. A. Hess W. M. Porter H. Jensen J. B.. (2004). Coronamycins, peptide antibiotics produced by a verticillate *Streptomyces* sp. (MSU-2110) endophytic on *Monstera* sp. Microbiololgy 150, 785–793. 10.1099/mic.0.26645-0 15073289

[B76] Feng W. W. Wang T. Bai J. L. Ding P. Xing K. Jiang J. H.. (2017). *Glutamicibacter halophytocola* sp. nov., an endophytic actinomycete isolated from the roots of a coastal halophyte, Limonium sinense. Int. J. Syst. Evol. Microbiol. 67, 1120–1125. 10.1099/ijsem.0.001775 28056223

[B77] Fiedler H. P. Bruntner C. Riedlinger J. Bull A. T. Knutsen G. Goodfellow M.. (2008). Proximicin, A., B and C, novel aminofuran antibiotic and anticancer compounds isolated from marine strains of the actinomycete *Verrucosispora*. J. Antibiot. 61, 158–163. 10.1038/ja.2008.125 18503194

[B78] Flowers T. J. Colmer T. D. (2015). Plant salt tolerance: adaptations in halophytes. Ann. Bot. 115, 327–331. 10.1093/aob/mcu267 25844430 PMC4332615

[B79] Fuentes M. S. Raimondo E. E. Amoroso M. J. Benimeli C. S. (2017). Removal of a mixture of pesticides by a *Streptomyces* consortium: influence of different soil systems. Chemosphere. 173, 359–367. 10.1016/j.chemosphere.2017.01.044 28126570

[B80] Furumai T. Yamakawa T. Yoshida R. Igarashi Y. (2003). Clethramycin, a new inhibitor of pollen tube growth with antifungal activity from *Streptomyces hygroscopicus* TP-A0623. I. Screening, taxonomy, fermentation, isolation and biological properties. J. Antibiot. 56, 700–704. 10.7164/antibiotics.56.700 14563159

[B81] Gangwar M. Dogra S. Gupta U. P. Kharwar R. N. (2014). Diversity and biopotential of endophytic actinomycetes from three medicinal plants in India. African. J. Microbiol. Res. 8, 184–191. 10.5897/AJMR2012.2452

[B82] Gauthier D. Diem H. G. Dommergues Y. (1981). *In vitro* nitrogen fixation by two actinomycete strains isolated from *Casuarina* nodules. Appl. Environ. Microbiol. 41, 306–308. 16345698 10.1128/aem.41.1.306-308.1981 PMC243679

[B83] Gayathri P. Muralikrishnan V. (2013). Isolation and characterization of endophytic actinomycetes from mangrove plant for antimicrobial activity. Int. J. Curr. Microbiol. Appl. Sci. 2, 78–89.

[B84] Gebhardt K. Schimana J. Krastel P. Dettner L. Rheinheimer J. Zeeck A.. (2002). Endophenazines, A.-D., new phenazine antibiotics from the arthropod associated endosymbiont *Streptomyces anulatus*. I. Taxonomy, fermentation, isolation and biological activities. J. Antibiot. 55, 794–800. 10.7164/antibiotics.55.794 12458768

[B85] Ghadin N. Zin N. M. Sabaratnam V. Badya N. Basri D. F. Lian H. H. (2008). Isolation and characterization of a novel endophytic *Streptomyces* SUK 06 with antimicrobial activity from Malaysian plant. Asian, J. Plant Sci. 7, 189–194. 10.3923/ajps.2008.189.194

[B86] Golinska P. Wypij M. Agarkar G. Rathod D. Dahm H. Rai M. (2015). Endophytic actinobacteria of medicinal plants: diversity and bioactivity. Antonie van Leeuwenhoek. 108, 267–289. 10.1007/s10482-015-0502-7 26093915 PMC4491368

[B87] González I. Ayuso-Sacido A. Anderson A. Genilloud O. (2005). Actinomycetes isolated from lichens: evaluation of their diversity and detection of biosynthetic gene sequences. FEMS Microbiol. Ecol. 54, 401–415. 10.1016/j.femsec.2005.05.004 16332338

[B88] Goodman A. A. (2014). Endophytic Actinomycetes as Potential Agents to Control Common Scab of Potatoes. All NMU Master's Theses. 32. Available online at: https://commons.nmu.edu/theses/32

[B89] Gos F. M. W. R. Savi D. C. Shaaban K. A. Thorson J. S. Aluizio R. Possiede Y. M. (2017). Antibacterial activity of endophytic actinomycetes isolated from the medicinal plant *Vochysia divergens* (Pantanal, Brazil). Front. Microbiol. 6:1642 10.3389/fmicb.2017.01642 PMC5592219 28932210

[B90] Goudjal Y. Toumatia O. Sabaou N. Barakate M. Mathieu F. Zitouni A. (2013). Endophytic actinomycetes from spontaneous plants of Algerian Sahara: indole-3-acetic acid production and tomato plants growth promoting activity. World J. Microbiol. Biotechnol. 29, 1821–1829. 10.1007/s11274-013-1344-y 23579766

[B91] Goudjal Y. Toumatia O. Yekkour A. Sabaoua N. Mathieuc F. Zitouni A. (2014). Biocontrol of *Rhizoctonia solani* damping-off and promotion of tomato plant growth by endophytic actinomycetes isolated from native plants of Algerian Sahara. Microbiol. Res. 169, 59–65. 10.1016/j.micres.2013.06.014 23920229

[B92] Govindasamy V. Franco C. M. M. Gupta V. V. S. R. (2014). Endophytic actinobacteria: Diversity and Ecology, in Advances in Endophytic Research, eds V. Verma, A. Gange (New Delhi: Springer), 27–59. 10.1007/978-81-322-1575-2_2

[B93] Gu Q. Liu N. Qiu D. H. Liu Z. H. Huang Y. (2006). Isolation, classification and antimicrobial activity of endophytic actinomycetes from plant leaves. Acta Microbiol. Sinica. 46, 778–782. 17172028

[B94] Guindon S. Gascuel O. (2003). A simple, fast, and accurate algorithm to estimate large phylogenies by maximum likelihood. Syst. Biol. 52, 696–704. 10.1080/10635150390235520 14530136

[B95] Gupta N. Mishra S. Basak U. C. (2009). Diversity of *Streptomyces* in mangrove ecosystem of Bhitarkanika. *Iran J*. Microbiol. 1, 37–42. Available online at: http://ijm.tums.ac.ir/index.php/ijm/article/view/28.26Jul.2018

[B96] Haase S. Neumann G. Kania A. Kuzyakov Y. Romheld V. Kandeler E. (2007). Elevation of atmospheric CO2 and N-nutritional status modify nodulation, nodule-carbon supply and root exudation of *Phaseolus vulgaris* L. Soil Biol. Biochem. 39, 2208–2221. 10.1016/j.soilbio.2007.03.014

[B97] Hallmann J. Quadt-Hallmann A. Mahaffee W. F. Kloepper J. W. (1997). Bacterial endophytes in agricultural crops. Can. J. Microbiol. 43, 895–914. 10.1139/m97-131

[B98] Hamedi J. Mohammadipanah F. Ventosa A. (2013). Systematic and biotechnological aspects of halophilic and halotolerant actinomycetes. Extremophiles 17, 1–13. 10.1007/s00792-012-0493-5 23129307

[B99] Han L. Huang X. S. Sattler I. Fu H. Z. Grabley S. Lin W. H. (2007). Two new constituents from mangrove *Bruguiera gymnorrhiza*. J. Asian Nat. Prod. Res. 9, 327–331. 10.1080/10286020600727574 17613617

[B100] Hardoim P. R. van-Overbeek L. S. van-Elsas J. D. (2008). Properties of bacterial endophytes and their proposed role in plant growth. Trends Microbiol. 16, 463–471. 10.1016/j.tim.2008.07.008 18789693

[B101] Hasegawa S. Meguro A. Shimizu M. Nishimura T. Kunoh H. (2006). Endophytic actinomycetes and their interactions with host plants. Actinomycetologica 20, 72–81. 10.3209/saj.20.72

[B102] Hasegawa T. Lechevalier M. P. Lechevalier H. A. (1978). New genus of the *Actinomycetales: Actinosynnema* gen. nov. *Int. J. Syst*. Bacteriol. 28, 304–310.

[B103] Hastuti R. D. D. Lenstari Y. Suwanto A. Saraswti R. (2012). *Endophytic Streptomyces* spp. as biocontrol agents of rice bacterial leaf blight pathogen (Xanthomonas oryzae pv. oryzae). HAYATI J. Biosci. 19, 155–162. 10.4308/hjb.19.4.155

[B104] Hata K. Sone K. (2008). Isolation of endophytes from leaves of *Neolitsea sericea* in broadleaf and conifer stands. Mycoscience. 49, 229–232. 10.1007/S10267-008-0411-Y

[B105] He H. Liu C. Zhao J. Li W. Pan T. Yang L.. (2014). *Streptomyces zhaozhouensis* sp. nov., an actinomycete isolated from Candelabra aloe (Aloe arborescens Mill). Int. J. Syst. Evol. Microbiol. 64, 1096–1101. 10.1099/ijs.0.056317-0 24368691

[B106] Hemalatha S. Rasool U. (2017). Marine endophytic actinomycetes assisted synthesis of copper nanoparticles (CuNPs): characterization and antibacterial efficacy against human pathogens. Mater. Lett. 194, 176–180. 10.1016/j.matlet.2017.02.055

[B107] Vazquez-Hernandez V. M. Ceapa C. D. Rodríguez-Luna S. D. Rodríguez-Sanoja R. Sánchez S. (2017). Draft genome sequence of *Streptomyces scabrisporus* NF3, an endophyte isolated from *Amphipterygium adstringens*. Genome Announc. 27, e00267–e00217. 10.1128/genomeA.00267-17 PMC5408122 28450524

[B108] Higashide E. Asai M. Ootsu K. Tanida S. Kozai Y. Hasegawa T. (1977). Ansamitocin, a group of novel maytansinoid antibiotics with antitumour properties from *Nocardia*. Nature. 270, 721–722. 10.1038/270721a0 593392

[B109] Huang H. Jiasen L. Yonghua H. Fang Z. Zhang K. Bao S. (2008). *Micromonospora rifamycinica* sp. nov., a novel actinomycete from mangrove sediment. Int. J. Syst. Evol. Microbiol. 58, 17–20. 10.1099/ijs.0.64484-0 18175675

[B110] Huang M. J. Rao M. P. Salam N. Xiao M. Huang H. Q. Li W. J. (2017). *Allostreptomyces psammosilenae* gen. nov., sp. nov., an endophytic actinobacterium isolated from the roots of *Psammosilene tunicoides* and emended description of the family *Streptomycetaceae* [Waksman and Henrici (1943) ^AL^] emend. Rainey et al. 1997, emend. Kim et al. 2003, emend. Zhi et al. 2009. Int. J. Syst. Evol. Microbiol. 67, 288–293. 10.1099/ijsem.0.001617 27902296

[B111] Huang X. L. Zhuang L. Lin H. P. Li J. Goodfellow M. Hong K. (2012). Isolation and bioactivity of endophytic filamentous actinobacteria from tropical medicinal plants. Afr. J. Biotechnol. 11, 9855–9864. 10.5897/AJB11.3839

[B112] Igarashi Y. (2004). Screening of novel bioactive compounds from plant associated actinomycetes. Actinomycetologica 18, 63–66. 10.3209/saj.18_63

[B113] Igarashi Y. Iida T. Yoshida R. Furumai T. (2002). Pteridic acids A and B, novel plant growth promoters with auxin-like activity from *Streptomyces hygroscopicus* TP-A0451. J. Antibiot. 55, 764–767. 10.7164/antibiotics.55.764 12374388

[B114] Igarashi Y. Iwashita T. Fujita T. Naoki H. Yamakawa T. Yoshida R.. (2003). Clethramycin, a new inhibitor of pollen tube growth with antifungal activity from *Streptomyces hygroscopicus* TP-A0623. II. Physico-chemical properties and structure determination. J. Antibiot. 56, 705–708. 10.7164/antibiotics.56.705 14563160

[B115] Igarashi Y. Miura S. S. Fujita T. Furumai T. (2006). Pterocidin, a cytotoxic compound from the endophytic *Streptomyces hygroscopicus*. J. Antibiot. 59, 193–195. 10.1038/ja.2006.28 16724461

[B116] Igarashi Y. Ogawa M. Sato Y. Saito N. Yoshida R. Kunoh H.. (2000). Fistupyrone, a novel inhibitor of the infection of Chinese cabbage by *Alternaria brassicicola*, from *Streptomyces* sp. TP-A0569. J. Antibiot. 53, 1117–1122. 10.7164/antibiotics.53.1117 11132956

[B117] Igarashi Y. Yanase S. Sugimoto K. Enomoto M. Miyanaga S. Trujillo M. E.. (2011). Lupinacidin, C., an inhibitor of tumor cell invasion from *Micromonospora lupini*. J. Nat. Prod. 74, 862–865. 10.1021/np100779t 21226490

[B118] Indananda C. Igarashi Y. Ikeda M. Oikawa T. Thamchaipenet A. (2013). Linfuranone, A., a new polyketide from plant-derived *Microbispora* sp. GMKU 363. J. Antibiot. 66, 675–677. 10.1038/ja.2013.67 23820612

[B119] Indananda C. Thamchaipenet A. Matsumoto A. Inahashi Y. Duangmal K. Takahashi Y. (2011). *Actinoallomurus oryzae* sp. nov., an endophytic actinomycete isolated from roots of a Thai jasmine rice plant. Int. J. Syst. Evol. Microbiol. 61, 737–741. 10.1099/ijs.0.022509-0 20418407

[B120] Inderiati S. Franco C. M. M. (2008). Isolation and identification of endophytic actinomycetes and their antifungal activity. J. Biotechnol. Res. 1, 1–6. ISSN: 1979-9756

[B121] Indraningrat A. A. Smidt H. Sipkema D. (2016). Bioprospecting sponge-associated microbes for antimicrobial compounds. Mar. Drugs. 14, 1–66. 10.3390/md14050087 27144573 PMC4882561

[B122] Jackson S. A. Crossman L. Almeida E. L. Margassery L. M. Kennedy J. Dobson A. D. W. (2018). Diverse and abundant secondary metabolism biosynthetic gene clusters in the genomes of marine sponge derived *Streptomyces* spp. isolates. Mar. Drugs 16, 1–18. 10.3390/md16020067 29461500 PMC5852495

[B123] Jain P. K. Joshi H. (2012). Coumarin: chemical and pharmacological profile. J. Appl. Pharm. Sci. 2, 236–240. 10.7324/JAPS.2012.2643

[B124] Janso J. E. Carter G. T. (2010). Biosynthetic potential of phylogenetically unique endophytic actinomycetes from tropical plants. Appl. Environ. Microbiol. 76, 4377–4386. 10.1128/AEM.02959-09 20472734 PMC2897433

[B125] Jensen S. I. Kuhl M. Prieme A. (2007). Different bacterial communities associated with the roots and bulk sediment of the seagrass *Zostera marina*. FEMS. Microbiol. Ecol. 62, 108–117. 10.1111/j.1574-6941.2007.00373.x 17825072

[B126] Jia F. Liu C. Wang X. Zhao J. Liu Q. Zhang J.. (2013). *Wangella harbinensis* gen. nov., sp. nov., a new member of the family Micromonosporaceae. Antonie van Leeuwenhoek 103, 399–408. 10.1007/s10482-012-9820-1 23011010

[B127] Jiang Z. K. Pan Z. Li F. N. Li X. J. Liu S. W. Tuo L.. (2017). *Marmoricola endophyticus* sp. nov., an endophytic actinobacterium isolated from the Spesia populnea. Int. J. Syst. Evol. Microbiol. 67, 4379–4384. 10.1099/ijsem.0.002297 28920827

[B128] Jiang Z. K. Tuo L. Huang D. L. Osterman I. A. Tyurin A. P. Liu S. W.. (2018). Diversity, novelty, and antimicrobial activity of endophytic actinobacteria from mangrove plants in Beilun Estuary National Nature Reserve of Guangxi, China. Front. Microbiol. 9:868. 10.3389/fmicb.2018.00868 29780376 PMC5945994

[B129] Jog R. Pandya M. Nareshkumar G. Rajkumar S. (2014). Mechanism of phosphate solubilization and antifungal activity of *Streptomyces* sp. isolated from wheat roots and rhizosphere and their application in improving plant growth. Microbiology 160, 778–788. 10.1099/mic.0.074146-0 24430493

[B130] Kaewkla O. Franco C. M. (2013). Rational approaches to improving the isolation of endophytic actinobacteria from Australian native trees. Microb. Ecol. 65, 384–393. 10.1007/s00248-012-0113-z 22976339

[B131] Kaewkla O. Thamchaipenet A. Franco C. M. (2017). *Micromonospora terminaliae* sp. nov., an endophytic actinobacterium isolated from the surface-sterilized stem of the medicinal plant Terminalia mucronata. Int. J. Syst. Evol. Microbiol. 225–230. 10.1099/ijsem.0.001600 28230521

[B132] Kamboj P. Gangwar M. Singh N. (2017). Scanning electron microscopy of endophytic actinomycete isolate against *Fusarium oxysporum* for various growth parameters on musk melon. Int. J. Curr. Microbiol. Appl. Sci. 6, 458–464. 10.20546/ijcmas.2017.611.054

[B133] Kandeler E. Mosier A. R. Morgan J. A. Milchunas D. G. King J. Y. Rudolph S. (2006). Response of soil microbial biomass and enzyme activities to the transient elevation of carbon dioxide in a semi-arid grassland. Soil Biol. Biochem. 38, 2448–2460. 10.1016/j.soilbio.2006.02.021

[B134] Khamna S. Yokota A. Lumyong S. (2009). Actinomycetes isolated from medicinal plant rhizosphere soils: diversity and screening of antifungal compounds, indole-3-acetic acid and siderophore production. World J. Microbiol. Biotechnol. 25, 649–655. 10.1007/s11274-008-9933-x

[B135] Khamna S. Yokota A. Peberdy J. F. Lumyong S. (2010). Indole-3-acetic acid production by *Streptomyces* sp. isolated from some Thai medicinal plant rhizosphere soils. EurAsia. J. Biosci. 4, 23–32. 10.5053/ejobios.2010.4.0.4

[B136] Khan N. Syed D. N. Ahmad N. Mukhtar H. (2013). Fisetin: a dietary antioxidant for health promotion. Antioxid. Redox Signal. 19, 151–162. 10.1089/ars.2012.4901 23121441 PMC3689181

[B137] Kim N. Shin J. C. Kim W. Hwang B. Y. Kim B. S. Hong Y. S.. (2006). Cytotoxic 6-alkylsalicylic acids from the endophytic *Streptomyces laceyi*. J. Antibiot. 59, 797–800. 10.1038/ja.2006.105 17323647

[B138] Komaki H. Ichikawa N. Hosoyama A. Fujita N. Thamchaipenet A. Igarashi Y. (2015). Draft genome sequence of Linfuranone producer *Microbispora* sp. GMKU 363. Genome Announc. 3:e01471–15. 10.1128/genomeA.01471-15 26659694 PMC4675959

[B139] Kominek L. A. (1972). Biosynthesis of novobiocin by *Streptomyces niveus*. Antimicrob. Agents Chemother. 1, 123–134. 10.1128/AAC.1.2.123 4680802 PMC444180

[B140] Kruasuwan W. Salih T. S. Brozio S. Hoskisson P. Thamchaipenet A. (2017). Draft genome sequence of plant growth-promoting endophytic *Streptomyces* sp. GKU 895 isolated from the roots of sugarcane. Genome Announc. 5, e00358–17. 10.1128/genomeA.00358-17 28495785 PMC5427220

[B141] Kuffner M. De Maria S. Puschenreiter M. Fallmann K. Wieshammer G. Gorfer M.. (2010). Culturable bacteria from Zn- and Cd-accumulating *Salix caprea* with differential effects on plant growth and heavy metal availability. J. Appl. Microbiol. 108, 1471–1484. 10.1111/j.1365-2672.2010.04670.x 20132372

[B142] Kulkarni-Almeida A. A. Brahma M. K. Padmanabhan P. Mishra P. D. Parab R. B. Gaikwad N. V.. (2011). Fermentation, isolation, structure, and antidiabetic activity of NFAT-133 produced by *Streptomyces* strain PM0324667. AMB Express. 1, 1–42. 10.1186/2191-0855-1-42 22104600 PMC3274447

[B143] Lee S. O. Choi G. J. Choi Y. H. Jang K. S. Park D. J. Kim C. J.. (2008). Isolation and characterization of endophytic actinomycetes from Chinese cabbage roots as antagonists to *Plasmodiophora brassicae*. J. Microbiol. Biotechnol. 18, 1741–1746. 10.4014/jmb.0800.108 19047815

[B144] Li F. L. Ma J. B. Mohamad O. A. Li S. H. Osman G. Li Y. Q. (2015). *Phytoactinopolyspora endophytica* gen. nov.sp. nov., a halotolerant filamentous actinomycetes isolated from the roots of Glycyrrhiza uralensis. Int. J. Syst. Evol. Microbiol. 65, 2671–2677. 10.1099/ijs.0.000322 25964514

[B145] Li F. N. Pan Z. Tuo L. Liu S. W. Zuo X. Chen L. (2017). Studies on the diversity and novelty of endophytic actinobacteria isolated from mangrove plants collected in Macao. Chin. J. Antibiot. 42, 26–34. 10.13461/j.cnki.cja.005906

[B146] Li J. Zhao G. Z. Chen H. H. Wang H. B. Qin S. Zhu W. Y. (2008). Antitumour and antimicrobial activities of endophytic *Streptomyces* from pharmaceutical plants in rainforest. Lett. Appl. Microbiol. 47, 574–580. 10.1111/j.1472-765X.2008.02470.x 19120929

[B147] Li J. Zhao G. Z. Huang H. Y. Qin S. Zhu W. Y. Zhao L. X.. (2012). Isolation and characterization of culturable endophytic actinobacteria associated with *Artemisia annua* L. Antonie van Leeuwenhoek. 101, 515–527. 10.1007/s10482-011-9661-3 22038129

[B148] Li L. Tang Y. L. Wei B. Xie Q. Y. Deng Z. Hong K. (2013). *Micromonospora sonneratiae* sp. *nov*., isolated from a root of Sonneratia apetala. Int. J. Syst. Evol. Microbiol. 63, 2383–2388. 10.1099/ijs.0.043570-0 23178729

[B149] Liu C. Wang X. Zhao J. Liu Q. Wang W. Guan X.. (2013). *Streptomyces harbinensis* sp. nov., an endophytic, ikarugamycin-producing actinomycete isolated from soybean root [Glycine max (L.) Merr.]. Int. J. Syst. Evol. Microbiol. 63, 3579–3584. 10.1099/ijs.0.050088-0 23584286

[B150] Liu S. W. Tuo L. Li X. J. Li F. N. Li J. Jiang M. G.. (2017). *Mangrovihabitans endophyticus* gen. nov., sp. nov., a new member of the family Micromonosporaceae isolated from Bruguiera sexangula. Int. J. Syst. Evol. Microbiol. 67, 1629–1636. 10.1099/ijsem.0.001764 28036245

[B151] Liu S. Xu M. Tuo L. Li X. Hu L. Chen L.. (2016). *Phycicoccus endophyticus* sp. nov., an endophytic actinobacterium isolated from Bruguiera gymnorhiza. Int. J. Syst. Evol. Microbiol. 66, 1105–1111. 10.1099/ijsem.0.000842 26653143

[B152] Liu Y. Jianwei Guo J. Li L. Asem M. D. Zhang Y. Mohamad O. A. (2017). Endophytic bacteria associated with endangered plant *Ferula sinkiangensis* K. M. Shen in an arid land: diversity and plant growth-promoting traits. J. Arid Land. 9, 432–445. 10.1007/s40333-017-0015-5

[B153] Lu C. Shen Y. (2007). A novel ansamycin, naphthomycin K from *Streptomyces* sp. J. Antibiot. 60, 649–653. 10.1038/ja.2007.84 17965482

[B154] Ma Y. Prasad M. N. Rajkumar M. Freitas H. (2011). Plant growth promoting rhizobacteria and endophytes accelerate phytoremediation of metalliferous soils. Biotechnol. Adv. 29, 248–258. 10.1016/j.biotechadv.2010.12.001 21147211

[B155] Ma Z. Zhao S. Cao T. Liu C. Huang Y. Gao Y.. (2016). *Verrucosispora sonchi* sp. nov., a novel endophytic actinobacterium isolated from the leaves of common sowthistle *(Sonchus oleraceus L.)*. Int. J. Syst. Evol. Microbiol. 66, 5430–5436. 10.1099/ijsem.0.001537 27707427

[B156] Madhaiyan M. Hu C. J. Kim S. J. Weon H. Y. Kwon S. W. Ji L. (2013). *Jatrophihabitans endophyticus* gen. nov., sp. nov., an endophytic actinobacterium isolated from a surface-sterilized stem of Jatropha curcas L. Int. J. Syst. Evol. Microbiol. 63, 1241–1248. 10.1099/ijs.0.039685-0 22798659

[B157] Maggini V. De Leo M. Mengoni A. Gallo E. R. Miceli E. Reidel R. V. B.. (2017). Plant-endophytes interaction influences the secondary metabolism in *Echinacea purpurea* (L.) Moench: an *in vitro* model. Sci Rep. 7:16924. 10.1038/s41598-017-17110-w 29208923 PMC5717142

[B158] Mansson M. Gram L. Larsen T. O. (2011). Production of bioactive secondary metabolites by marine *vibrionaceae*. Mar. Drugs. 9, 1440–1468. 10.3390/md9091440 22131950 PMC3225927

[B159] Masand M. Jose P. A. Menghani E. Jebakumar S. R. D. (2015). Continuing hunt for endophytic actinomycetes as a source of novel biologically active metabolites. World J. Microbiol. Biotechnol. 31, 1863–1875. 10.1007/s11274-015-1950-y 26410426

[B160] Meguro A. Hasegawa S. Nishimura T. Kunoh H. (2006). Salt tolerance in tissue-cultured seedlings of mountain laurel enhanced by endophytic colonization of *Streptomyce*s *padanus* AOK-30, Abst. 2006 Annu. Meeting of Soc. Actinomycetes Japan, p. 97, 2006 (in Japanese).

[B161] Mendes R. Garbeva P. Raaijmakers J. M. (2013). The rhizosphere microbiome: significance of plant beneficial, plant pathogenic, and human pathogenic microorganisms. FEMS Microbiol. Rev. 37, 634–663. 10.1111/1574-6976.12028 23790204

[B162] Mesta S. C. Onkarappa R. Chittara S. Dinesh J. Chaitra K. (2017). Screening of antimicrobial and antioxidant activities of endophytic actinomycetes isolated from *Rhizophora mucronata* and *Sonneratia caseolaris*. Int. J. Pharm. Biol. Sci. Arch. 8, 22–28.

[B163] Miller K. I. Qing C. Sze D. M. Roufogalis B. D. Neilan B. A. (2012). Culturable endophytes of medicinal plants and the genetic basis for their bioactivity. Microb. Ecol. 64, 431–449. 10.1007/s00248-012-0044-8 22430508

[B164] Minamiyama H. Shimizu M. Kunoh H. Furumai T. Igarashi Y. Onaka H. (2003). Multiplication of isolate R-5 of *Streptomyces galbus* on rhododendron leaves and its production of cell wall-degrading enzymes. J. Gen. Plant Pathol. 69, 65–70. 10.1007/s10327-002-0014-y

[B165] Mingma R. Pathom-aree W. Trakulnaleamsai S. Thamchaipenet A. Duangmal K. (2014). Isolation of rhizospheric and roots endophytic actinomycetes from *Leguminosae* plant and their activities to inhibit soybean pathogen, *Xanthomonas campestris* pv. glycine. World J. Microbiol. Biotechnol. 30, 271–280. 10.1007/s11274-013-1451-9 23913026

[B166] Mishra S. K. Taft W. H. Putnam A. R. Ries S. K. (1987). Plant growth regulatory metabolites from novel actinomycetes. J. Plant Growth Regul. 6, 75–84. 10.1007/BF02026457

[B167] Mitter B. Petric A. Shin M. W. Chain P. S. G. Hauberg- Lotte L. Reinhold-Hurek B.. (2013). Comparative genome analysis of *Burkholderia phytofirmans* PsJN reveals a wide spectrum of endophytic lifestyles based on interaction strategies with host plants. Front. Plant Sci. 4:120. 10.3389/fpls.2013.00120 23641251 PMC3639386

[B168] Mohammadipanah F. Wink J. (2016). Actinobacteria from arid and desert habitats: diversity and biological activity. Front. Microbiol. 6:1541. 10.3389/fmicb.2015.01541 26858692 PMC4729944

[B169] Nair D. N. Padmavathy S. (2014). Impact of endophytic microorganisms on plants, environment and humans. Sci. World J. 2014:250693. 10.1155/2014/250693 24587715 PMC3920680

[B170] Nalini M. S. Prakash H. S. (2017). Diversity and bioprospecting of actinomycete endophytes from the medicinal plants. Lett. Appl. Microbiol. 64, 261–270. 10.1111/lam.12718 28107573

[B171] Nautiyal C. S. Bhadauria S. Kumar P. Lal H. Mondal R. Verma D. (2000). Stress induced phosphate solubilization in bacteria isolated from alkaline soils. FEMS Microbiol. Lett. 182, 291–296. 10.1016/S0378-1097(99)00605-9 10620681

[B172] Nimnoi P. Pongsilp N. Lumyong S. (2010). Endophytic actinomycetes isolated from *Aquilaria crassna* Pierre ex. Lec and screening of plant growth-promoters production. World J. Microbiol. Biotechnol. 26, 193–203. 10.1007/s11274-009-0159-3

[B173] Nishimura T. Meguro A. Hasegawa S. Nakagawa Y. Shimizu M. Kunoh H. (2002). An endophytic actinomycete, *Streptomyces* sp. AOK-30, isolated from mountain laurel and its antifungal activity. J. Gen. Plant Pathol. 68, 390–397. 10.1007/PL00013109

[B174] Okazaki T. (2003). Studies on actinomycetes isolated from plant leaves, in Selective Isolation of rare Actinomycetes, ed Kurtböke I. I (Canberra: National Library of Australia), 102–122.

[B175] Palaniyandi S. Yang S. H. Damodharan K. Suh J. W. (2013). Genetic and functional characterization of culturable plant-beneficial actinobacteria associated with yam rhizosphere. J. Basic Microbiol. 53, 985–995. 10.1002/jobm.201200531 23681763

[B176] Panche A. N. Diwan A. D. Chandra S. R. (2016). Flavonoids: an overview. J. Nutr. Sci. 5, 1–15. 10.1017/jns.2016.41 28620474 PMC5465813

[B177] Passari A. K. Chandra P. Zothanpuia Mishra V. K. Leo V. V. Gupta V. K.. (2016). Detection of biosynthetic gene and phytohormone production by endophytic actinobacteria associated with *Solanum lycopersicum* and their plant-growth-promoting effect. Res. Microbiol. 167, 692–705. 10.1016/j.resmic.2016.07.001 27421813

[B178] Passari A. K. Mishra V. K. Gupta V. K. Yadav M. K. Saikia R. Singh B. P. (2015a). *In vitro* and *in vivo* plant growth promoting activities and DNA fingerprinting of antagonistic endophytic actinomycetes associates with medicinal plants. PLoS ONE 10:e0139468. 10.1371/journal.pone.0139468 26422789 PMC4589368

[B179] Passari A. K. Mishra V. K. Saikia R. Gupta V. K. Singh B. P. (2015b). Isolation, abundance and phylogenetic affiliation of endophytic actinomycetes associated with medicinal plants and screening for their in vitro antimicrobial biosynthetic potential. Front. Microbiol. 6:e00273. 10.3389/fmicb.2015.00273 25904906 PMC4388002

[B180] Passari A. K. Mishra V. K. Singh G. Singh P. Kumar B. Gupta V. K.. (2018). Insights into the functionality of endophytic actinobacteria with a focus on their biosynthetic potential and secondary metabolites production. Sci. Rep. 8:4650. 10.1038/s41598-018-22947-w 29531340 PMC5847523

[B181] Philippot L. Raaijmakers J. M. Lemanceau P. van der Putten W. H. (2013). Going back to the roots: the microbial ecology of the rhizosphere. Nat. Rev. Microbiol. 11, 789–799. 10.1038/nrmicro3109 24056930

[B182] Phuakjaiphaeo C. Kunasakdakul K. (2015). Isolation and screening for inhibitory activity on *Alternaria brassicicola* of endophytic actinomycetes from *Centella asiatica* (L.) Urban. J. Agri. Technol. 11, 903–912.

[B183] Pimentel M. R. Molina G. Dionisio A. P. Maróstica M. R. Pastore G. M. (2011). Use of endophytes to obtain bioactive compounds and their application in biotransformation process. Biotechnol. Res. Int. 2011:576286. 10.4061/2011/576286 21350663 PMC3042614

[B184] Prakash O. Nimonkar Y. Munot H. Sharma A. Vemuluri V. R. Chavadar M. S.. (2014). Description of *Micrococcus aloeverae* nov., an endophytic actinobacterium isolated from Aloe vera. Int. J. Syst. Evol. Microbiol. 64, 3427–3433. 10.1099/ijs.0.063339-0 25048212

[B185] Prashar P. Shah S. (2016). Impact of fertilizers and pesticides on soil microflora in agriculture. Sustainable Agriculture Reviews. 19, 331–362. 10.1007/978-3-319-26777-7_8

[B186] Pujiyanto S. Lestari Y. Suwanto A. Budiarti S. Darusman K. L. (2012). Alpha-glucosidase inhibitor activity and characterization of endophytic actinomycetes isolated from some Indonesian diabetic medicinal plants. Int. J. Pharm. Pharm. Sci. 4, 975–1491.

[B187] Pullen C. Schmitz P. Meurer K. Bamberg D. D. Lohmann S. De Castro Franca S.. (2002). New and bioactive compounds from *Streptomyces* strains residing in the wood of *Celastraceae*. Planta 216, 162–167. 10.1007/s00425-002-0874-6 12430026

[B188] Purushotham N. Jones E. Monk J. Ridgway H. (2018). Community Structure of endophytic actinobacteria in a New Zealand native medicinal plant *Pseudowintera colorata* (Horopito) and their influence on plant growth. Microb. Ecol. 1153–1159. 10.1007/s00248-018-1153-9 29435598

[B189] Qin S. Bian G. K. Tamura T. Zhang Y. J. Zhang W. D. Cao C. L.. (2013b). *Streptomyces halophytocola* sp. nov., an endophytic actinomycete isolated from the surface-sterilized stems of a coastal halophyte *Tamarix chinensis* Lour. Int. J. Syst. Evol. Microbiol. 63, 2770–2775. 10.1099/ijs.0.047456-0 23291896

[B190] Qin S. Bian G. K. Zhang Y. J. Xing K. Cao C. L. Liu C. H.. (2013a). *Modestobacter roseus* sp. nov., an endophytic actinomycete isolated from the coastal halophyte *Salicornia europaea* Linn, and emended description of the genus *Modestobacter*. Int. J. Syst. Evol. Microbiol. 63, 2197–2202. 10.1099/ijs.0.044412-0 23148095

[B191] Qin S. Chen H. H. Zhao G. Z. Li J. Zhu W. Y. Xu L. H.. (2012a). Abundant and diverse endophytic actinobacteria associated with medicinal plant *Maytenus austroyunnanensis* in Xishuangbanna tropical rainforest revealed by culture-dependent and culture-independent methods. Environ. Microbiol. Rep. 4, 522–531. 10.1111/j.1758-2229.2012.00357.x 23760897

[B192] Qin S. Feng W. W. Wang T. T. Ding P. Xing K. Jiang J. H. (2017). Plant growth-promoting effect and genomic analysis of the beneficial endophyte *Streptomyces* sp. KLBMP 5084 isolated from halophyte *Limonium sinense*. Plant Soil. 416, 117–132. 10.1007/s11104-017-3192-2

[B193] Qin S. Fenga W. W. Xing K. Baia J. L. Yuana B. Liu W. J. (2015). Complete genome sequence of *Kibdelosporangium phytohabitans* KLBMP 1111^T^, a plant growth promoting endophytic actinomycetes isolated from oil-seed plant *Jatropha curcas* L. J. Biotechnol. 20, 129–130. 10.1016/j.jbiotec.2015.10.017 26516119

[B194] Qin S. Li J. Chen H. H. Zhao G. Z. Zhu W. Y. Jiang C. L.. (2009). Isolation diversity and antimicrobial activity of rare actinobacteria from medicinal plants of tropical rain forests in Xishuangbanna, China. Appl. Environ. Microbiol. 75, 6176–6186. 10.1128/AEM.01034-09 19648362 PMC2753051

[B195] Qin S. Xing K. Jiang J. H. Xu L. H. Li W. J. (2011). Biodiversity, bioactive natural products and biotechnological potential of plant-associated endophytic actinobacteria. Appl. Microbiol. Biotechnol. 89, 457–473. 10.1007/s00253-010-2923-6 20941490

[B196] Qin S. Yuan B. Zhang Y. J. Bian G. K. Tamura T. Sun B. Z.. (2012b). *Nocardioides panzhihuaensis* sp. nov., a novel endophytic actinomycete isolated from medicinal plant Jatropha curcas L. Antonie van Leeuwenhoek. 102, 353–360. 10.1007/s10482-012-9745-8 22552630

[B197] Qiu P. Feng Z. X. Tian J. W. Lei Z. C. Wang L. Zeng Z. G.. (2015). Diversity, bioactivities, and metabolic potentials of endophytic actinomycetes isolated from traditional medicinal plants in Sichuan, China. Chin. J. Nat. Med. 13, 942–953. 10.1016/S1875-5364(15)30102-3 26721714

[B198] Rajivgandhi G. Ramachandran G. Muruthupandy M. Saravanakumar S. Manoharan N. Viji R. (2018). Antibacterial effect of endophytic actinomycetes from marine algae against multi drug resistant Gram negative bacteria. *Examines in Mar. Biol*. Oceanogr. 1, 1–8.

[B199] Rajivgandhi G. Vijayan R. Kannan M. Santhanakrishnan M. Manoharan N. (2016). Molecular characterization and antibacterial effect of endophytic actinomycetes *Nocardiopsis* sp. GRG1 (KT235640) from brown algae against MDR strains of uropathogens. Bioactive Mater. 1, 140–150. 10.1016/j.bioactmat.2016.11.002 29744403 PMC5883993

[B200] Rajkumar M. Ae N. Freitas H. (2009). Endophytic bacteria and their potential to enhance heavy metal phytoextraction. Chemosphere 77, 153–160. 10.1016/j.chemosphere.2009.06.047 19647283

[B201] Rajkumar M. Nagendran R. Lee K. J. Lee W. H. Kim S. Z. (2006). Influence of plant growth promoting bacteria and Cr6+ on the growth of Indian mustard. Chemosphere 62, 741–748. 10.1016/j.chemosphere.2005.04.117 15982703

[B202] Remali J. Sarmin N. M. Ng C. L. Tiong J. J. L. Aizat W. M. Keong L. K.. (2017). Genomic characterization of a new endophytic *Streptomyces kebangsaanensis* identifies biosynthetic pathway gene clusters for novel phenazine antibiotic production. Peer J. 5:e3738. 10.7717/peerj.3738 29201559 PMC5712208

[B203] Roh H. Uguru G. C. Ko H. J. Kim S. Kim B. Y. Goodfellow M. (2011). J. Bacteriol. 193, 3391–3392. 10.1128/JB.05041-11 21551311 PMC3133294

[B204] Rosenblueth M. Martínez-Romero E. (2006). Bacterial endophytes and their interactions with hosts. Mol. Plant Microbe Interact. 19, 827–837. 10.1094/MPMI-19-0827 16903349

[B205] Rungin S. Indananda C. Suttiviriya P. Kruasuwan W. Jaemsaeng R. Thamchaipenet A. (2012). Plant growth enhancing effects by a siderophore-producing endophytic *streptomycete* isolated from a Thai jasmine rice plant *(Oryza sativa* L. cv. KDML105). Antonie van Leeuwenhoek 102, 463–472. 10.1007/s10482-012-9778-z 22836676

[B206] Sabaou N. Bounaga N. Bounaga D. (1983). Actions antibiotique, mycolytique et parasitaire de deux actinomycètes envers *Fusarium oxysporum f*. sp. albedinis et autres formae specials. Can. J. Microbiol. 29, 194–199. 10.1139/m83-033

[B207] Salam N. Devi A. M. Li W. J. (2016). Direct plant growth-promoting ability of actinobacteria in grain legumes, in Plant Growth Promoting Actinobacteria eds Subramaniam G. Arumugam S. Rajendran V. (Singapore: Springer), 1–16. 10.1007/978-981-10-0707-1_1

[B208] Salam N. Khieu T. N. Liu M. J. Vu T. T. Ky S. C. Quach N. T.. (2017). Endophytic actinobacteria associated with *Dracaena cochinchinensis* Lour: isolation, diversity, and their cytotoxic activities. BioMed. Res. Int. 2017, 1–11. 10.1155/2017/1308563 28484706 PMC5397652

[B209] Sasaki T. Igarashi Y. Ogawa M. Furumai T. (2002). Identification of 6- prenylindole as an antifungal metabolite of *Streptomyces* sp. TP- A0595 and synthesis and bioactivity of 6-substituted indoles. J. Antibiot. 55, 1009–1012. 10.1002/chin.200319203 12546422

[B210] Sasaki T. Igarashi Y. Saito N. Furumai T. (2001). Cedarmycins, A., and B, new antimicrobial antibiotics from *Streptomyces* sp. TP-A0456. J. Antibiot. 54, 567–572. 10.7164/antibiotics.54.567 11560375

[B211] Savi D. C. Haminiuk C. W. Sora G. T. S. Adamoski D. M. Kenski J. Winnischofer S. M. B. (2015a). Antitumor, antioxidant and antibacterial activities of secondary metabolites extracted by endophytic actinomycetes isolated from *Vochysia divergens*. Int. J. Pharm. Chem. Biol. Sci. 5, 347–356.

[B212] Savi D. C. Shaaban K. A. Ponomareva L. V. Possiede Y. M. Thorson J. S. Glienke C.. (2015b). *Microbispora* sp. LGMB259 endophytic actinomycete isolated from *Vochysia divergens* (Pantanal, Brazil) producing β-carbolines and indoles with biological activity. Curr. Microbiol. 70, 345–354. 10.1007/s00284-014-0724-3 25385358 PMC4318781

[B213] Schenk P. M. Carvalhais L. C. Kazan K. (2012). Unraveling plant microbe interactions: can multi-species transcriptomics help? Trends Biotecnol. 30, 177–184. 10.1016/j.tibtech.2011.11.002 22209623

[B214] Sculthorpe C. D. (1985). The Biology of Aquatic Vascular Plants. London: Edward Arnold press.

[B215] Sessitsch A. Kuffner M. Kidd P. Vangronsveld J. Wenzel W. W. Fallmann K.. (2013). The role of plant-associated bacteria in the mobilization and phytoextraction of trace elements in contaminated soils. Soil Biol. Biochem. 60, 182–194. 10.1016/j.soilbio.2013.01.012 23645938 PMC3618436

[B216] Sgroy V. Cassan F. Masciarelli O. Del Papa M. F. Luna V. (2009). Isolation and characterization of endophytic plant-growth promoting or stress homeostasis-regulating bacteria associated to the halophyte *Prosopis strombulifer*a. Appl. Microbiol. Biotechnol. 85, 371–381. 10.1007/s00253-009-2116-3 19655138

[B217] Shekhar N. Bhattacharya D. Kumar D. Gupta R. K. (2006). Biocontrol of wood-rotting fungi with *Streptomyces violaceusniger* XL-2. Can. J. Microbiol. 52, 805–808. 10.1139/w06-035 17110971

[B218] Shen Y. Liu C. Wang X. Zhao J. Jia F. Zhang Y. (2013). *Actinoplanes hulinensis* sp nov., a novel actinomycete isolated from soybean root (*Glycine max* (L.) Merr). Antonie van Leeuwenhoek. 103, 293–298. 10.1007/s10482-012-9809-9 23114571

[B219] Shimizu M. Igarashi Y. Furumai T. Onaka H. Kunoh H. (2004). Identification of endophytic *Streptomyces* sp. R-5 and analysis of its antimicrobial metabolites. J. Gen. Plant Pathol. 70, 66–68. 10.1007/s10327-003-0082-7

[B220] Shimizu M. Nakagawa Y. Sato Y. Furumai T. Igarashi Y. Onaka H. (2000). Studies on endophytic actinomycetes (I). *Streptomyces sp*. isolated from Rhododendron and its antifungal activity. J. Gen. Plant Pathol. 66, 360–366. 10.1007/PL00012978

[B221] Shimizu M. Yazawa S. Ushijima Y. (2009). A promising strain of endophytic *Streptomyces* sp. for biological control of cucumber anthracnose. J. Gen. Plant Pathol. 75, 27–36. 10.1007/s10327-008-0138-9

[B222] Shinwari M. M. Alharbi S. A. Ara I. Wainwright M. (2013). Evaluation of antibiotic producing genes in Streptomyces isolated from a desert environment of Saudi Arabia. Life Sci. J. 10, 974–980.

[B223] Shutsrirung A. Chromkaew Y. Pathom-Aree W. Choonluchanon S. Boonkerd N. (2013). Diversity of endophytic actinomycetes in mandarin grown in northern Thailand, their phytohormone production potential and plant growth promoting activity. Soil Sci. Plant Nutr. 59, 322–330. 10.1080/00380768.2013.776935

[B224] Singh M. J. Sedhuraman P. (2015). Biosurfactant, polythene, plastic, and diesel biodegradation activity of endophytic *Nocardiopsis* sp. mrinalini9 isolated from *Hibiscus rosasinensis* leaves. Bioresour. Bioprocess. 2, 1–7. 10.1186/s40643-014-0034-4

[B225] Singh R. Dubey A. K. (2015). Endophytic actinomycetes as emerging source for therapeutic compounds. Indo Global, J. Pharm. Sci. 5, 106–116. 10.1038/ja.2017.20

[B226] Singh S. P. Gaur R. (2016). Evaluation of antagonistic and plant growth promoting activities of chitinolytic endophytic actinomycetes associated with medicinal plants against *Sclerotium rolfsii* in chickpea. J. Appl. Microbiol. 121, 506–518. 10.1111/jam.13176 27170067

[B227] Siyu-Mao Hong-Chen Li-Chen Chuanxi-Wang Wei-Jia Xiaoming-Chen. (2013). Two novel ansamitocin analogs from *Actinosynnema pretiosum*. Nat. Prod. Res. 27, 1532–1536. 10.1080/14786419.2012.733388 23061718

[B228] Spalding M. Blasco F. Field C. (eds.). (1997). World Mangrove Atlas: International Society for Mangrove Ecosystems. Okinawa, 43–178. Available online at: http://www.archive.org/details/worldmangroveatl97spal

[B229] Sreeja S. J. Gopal K. S. (2013). Bio-efficacy of endophytic actinomycetes for plant growth promotion and management of bacterial wilt in tomato. Pest Manag. Hort. Ecosyst. 19, 63–66.

[B230] Stamford T. L. M. Stamford N. P. Coelho L. C. B. B. Araujo J. M. (2002). Production and characterization of a thermostable glucoamylase from *Streptosporangium* sp. endophyte of maize leaves. Bioresour. Technol. 83, 105–109. 10.1016/S0960-8524(01)00206-1 12056484

[B231] Stepniewska Z. Kuzniar A. (2013). Endophytic microorganisms- promising applications in bioremediation of greenhouse gases. Appl. Microbiol. Biotechnol. 97, 9589–9596. 10.1007/s00253-013-5235-9 24048641 PMC3825493

[B232] Strobel G. A. Daisy B. (2003). Bioprospecting for microbial endophytes and their natural products. Microbiol. Mol. Biol. Rev. 67, 491–502. 10.1128/MMBR.67.4.491-502.2003 14665674 PMC309047

[B233] Subbulakshmi G. K. Thalavaipandian A. Bagyalakshmi R. V. Rajendran A. (2012). Bioactive endophytic fungal isolates of *Biota orientalis* (L) Endl, *Pinus excelsa* wall and *Thuja occidentalis* L. Int. J. Adv. Life Sci. 4, 9–15.

[B234] Subramani R. Aalbersberg W. (2013). Culturable rare actinomycetes: diversity, isolation and marine natural product discovery. Appl. Microbiol. Biotechnol. 97, 9291–9321. 10.1007/s00253-013-5229-7 24057404

[B235] Sun C. H. Li F. N. Cai Z. Wu L. (2017). Brief introduction of mangrove researches including drug discovery granted by National Natural Science Foundation of China from 1986 to 2016. Chin. J. Antibiot. 42, 241–248. 10.13461/j.cnki.cja.005904

[B236] Sun J. Cardoza V. Mitchell D. M. Bright L. Oldroyd G. Harris J. M. (2006). Crosstalk between jasmonic acid, ethylene and Nod factor signaling allows integration of diverse inputs for regulation of nodulation. Plant J. 46, 961–970. 10.1111/j.1365-313X.2006.02751.x 16805730

[B237] Taechowisan T. Chanaphat S. Ruensamran W. Phutdhawong W. S. (2014). Antibacterial activity of new flavonoids from *Streptomyces* sp. BT01; an endophyte in *Boesenbergia rotunda* (L.) Mansf. J. Appl. Pharm. Sci. 4, 8–13. 10.7324/JAPS.2014.40402

[B238] Taechowisan T. Chuaychot N. Chanaphat S. Shen Y. (2009). Anti-oxidative and inhibitory activity on nitric oxide production of flavonoids from *Streptomyces* sp. Tc052. J. Pharm. Res. 2, 313–316.

[B239] Taechowisan T. Chuaychot N. Chanaphat S. Wanbanjob A. Shen Y. (2008). Biological activity of chemical constituents isolated from *Streptomyces* sp. Tc052, an endophyte in Alpinia galanga. Int. J. Pharmacol. 4, 95–101. 10.3923/ijp.2008.95.101

[B240] Taechowisan T. Lu C. Shen Y. Lumyong S. (2007). Antitumor activity of 4-arylcoumarins from endophytic *Streptomyces aureofaciens* CMUAc130. J. Cancer Res. Ther. 3, 86–91. 10.4103/0973-1482.34685 17998729

[B241] Taechowisan T. Peberdy J. F. Lumyong S. (2003). Isolation of endophytic actinomycetes from selected plants and their antifungal activity. World, J. Microbiol. Biotechnol. 19, 381–385. 10.1023/A:1023901107182

[B242] Taechowisan T. Wanbanjob A. Tuntiwachwuttikul P. Taylor W. C. (2006). Identification of *Streptomyces* sp. Tc022, an endophyte in *Alpinia galanga*, and the isolation of actinomycin D. Ann. Microbiol. 56, 113–117. 10.1007/BF03174991

[B243] Tang S. K. Li W. J. Dong W. Zhang Y. G. Xu L. H. (2003). Studies of the biological characteristics of some halophilic and halotolerant actinomycetes isolated from saline and alkaline soils. Actinomycetologica 17, 6–10. 10.3209/saj.17_6

[B244] Tanvir R. Sajid I. Hasnain S. (2014). Larvicidal potential of *Asteraceae* family endophytic actinomycetes against *Culex quinquefasciatus* mosquito larvae. Nat. Prod. Res. 28, 2048–2052. 10.1080/14786419.2014.919579 24865275

[B245] Tanvir R. Sajid I. Hasnain S. Kulik A. Gron S. (2016). Rare actinomycetes *Nocardia caishijiensis* and *Pseudonocardia carboxydivorans* as endophytes; their bioactivity and metabolites evaluation. Microbiol. Res. 185, 22–35. 10.1016/j.micres.2016.01.003 26946375

[B246] Thamchaipenet A. Indananda C. Bunyoo C. Duangmal K. Matsumoto A. Takahashi Y. (2010). *Actinoallomurus acaciae* sp. nov., an endophytic actinomycete isolated from Acacia auriculiformis, A. Cunn. ex Benth. Int. J. Syst. Evol. Microbiol. 60, 554–559. 10.1099/ijs.0.012237-0 19654340

[B247] Thao P. T. H. Mai-Linh N. V. Hong-Lien N. T. Hieu N. V. (2016). Biological characteristics and antimicrobial activity of endophytic *Streptomyces* sp. TQR12- 4 isolated from elite *Citrus nobilis* cultivar Ham Yen of Vietnam Phan. Int. J. Microbiol. 2016:7207818 10.1155/2016/7207818 27795709 PMC5067319

[B248] Thawai C. (2015). *Micromonospora costi* sp. nov., isolated from a leaf of Costus speciosus. Int. J. Syst. Evol. Microbiol. 65, 1456–1461. 10.1099/ijs.0.000120 25687348

[B249] Tholl D. (2015). Biosynthesis and biological functions of terpenoids in plants. Adv. Biochem. Eng. Biotechnol. 148, 63–106. 10.1007/10_2014_295 25583224

[B250] Thumar J. T. Dhulia K. Singh S. P. (2010). Isolation and partial purification of an antimicrobial agent from halotolerant alkaliphilic *Streptomyces aburaviensis* strain Kut-8. World J. Microbiol. Biotechnol. 26, 2081–2087. 10.1007/s11274-010-0394-7

[B251] Tian X. Cao H. Tan W. Han M. Chen Y. Liu Y.. (2007). Diversity of cultivated and uncultivated actinobacterial endophytes in the stems and roots of rice. Microb. Ecol. 53, 700–707. 10.1007/s00248-006-9163-4 17334856

[B252] Ting A. S. Y. Hermanto A. Peh K. L. (2014). Indigenous actinomycetes from empty fruit bunch compost of oil palm: evaluation on enzymatic and antagonistic properties. Biocatal. Agr. Biotechnol. 3, 310–315. 10.1016/j.bcab.2014.03.004

[B253] Tiwari P. Kumar B. Kaur M. Kaur G. Kaur H. (2011). Phytochemical screening and extraction: a review. Int. Pharm. Sci. 1 98–105.

[B254] Toumatia O. Compant S. Yekkour A. Goudjal Y. Sabaou N. Mathieu F. (2016). Biocontrol and plant growth promoting properties of *Streptomyces mutabilis* strain IA1 isolated from a Saharan soil on wheat seedlings and visualization of its niches of colonization. S. Afr. J. Bot. 105, 234–239. 10.1016/j.sajb.2016.03.020

[B255] Trujillo M. E. Kroppenstedt R. M. Fernández-Molinero C. Schumann P. Martínez-Molina E. (2007). *Micromonospora lupini* sp. nov. and Micromonospora saelicesensis sp. nov., isolated from root nodules of Lupinus angustifolius. Int. J. Syst. Evol. Microbiol. 57, 2799–2804. 10.1099/ijs.0.65192-0 18048727

[B256] Tuntiwachwuttikul P. Taechowisan T. Wanbanjob A. Thadaniti S. (2008). Lansai, A.-D., secondary metabolites from *Streptomyces* sp. SUC1. Tetrahedron 64, 7583–7586. 10.1016/j.tet.2008.05.104

[B257] Turner T. R. James E. K. Poole P. S. (2013). The plant microbiome. Genome Biol. 14:209. 10.1186/gb-2013-14-6-209 23805896 PMC3706808

[B258] Van der Hiejden M. G. A. Bardgett R. D. van Straalen N. M. (2008). The unseen majority: soil microbes as drivers of plant diversity and productivity in terrestrial ecosystems. Ecol. Lett. 11, 296–310. 10.1111/j.1461-0248.2007.01139.x 18047587

[B259] Vandeputte O. Oden S. Mol A. Vereecke D. Goethals K. El Jaziri M.. (2005). Biosynthesis of auxin by the Gram-positive phytopathogen *Rhodococcus fascians* is controlled by compounds specific to infected plant tissues. Appl. Environ. Microbiol. 71, 1169–1177. 10.1128/AEM.71.3.1169-1177.2005 15746315 PMC1065166

[B260] Verma V. C. Singh S. K. Prakash S. (2011). Biocontrol and plant growth promotion potential of siderophore producing endophytic Streptomyces from *Azadirachta indica* A. Juss. J. Basic Microbiol. 51, 550–556. 10.1002/jobm.201000155 21656792

[B261] Villamil S. F. Stoppani A. O. M. Dubin M. (2004). Redox cycling of β-Lapachone and structural analogues in microsomal and cytosol liver preparations. Methods Enzymol. 378, 67–87. 10.1016/S0076-6879(04)78004-0 15038958

[B262] Viterbo A. Landau U. Kim S. Chernin L. Chet I. (2010). Characterization of ACC deaminase from the biocontrol and plant growth-promoting agent *Trichoderma asperellum* T203. FEMS Microbiol. Lett. 305, 42–48. 10.1111/j.1574-6968.2010.01910.x 20148973

[B263] Wan M. Li G. Zhang J. Jiang D. Huang H. C. (2008). Effect of volatile substances of *Streptomyces platensis* F-1 on control of plant fungal diseases. Biol. Control. 46, 552–559. 10.1016/j.biocontrol.2008.05.015

[B264] Wang H. F. Li L. Zhang Y. G. Hozzein W. N. Zhou X. K. Liu W. H. (2015d). *Arthrobacter endophyticus* sp. nov., a novel endophytic actinobacterium isolated from root of Salsola affinis (C. A. Mey). Int. J. Syst. Evol. Microbiol. 65, 2154–2160. 10.1099/ijs.0.000235 25858247

[B265] Wang H. F. Zhang Y. G. Chen J. Y. Guo J. W. Li L. Hozzein W. N.. (2015a). *Frigoribacterium endophyticum* sp. nov., an endophytic actinobacterium isolated from the root of Anabasis elatior (C. A. Mey.) Schischk. Int. J. Syst. Evol. Microbiol. 65. 1207–1212. 10.1099/ijs.0.000081 25609679

[B266] Wang H. F. Zhang Y. G. Cheng J. Hozzein W. N. Liu W. H. Li L.. (2015b). *Labedella endophytica* sp. nov., a novel endophytic actinobacterium isolated from stem of Anabasis elatior (C. A. Mey.) Schischk. Int. J. Syst. Evol. Microbiol. 107, 95–102. 10.1007/s10482-014-0307-0 25331338

[B267] Wang H. F. Zhang Y. G. Li L. Liu W. H. Hozzein W. N. Chen J. Y.. (2015c). *Okibacterium endophyticum* sp. nov., a novel endophytic actinobacterium isolated from roots of Salsola affinis C. A. Mey. Antonie van Leeuwenhoek. 107, 835–843. 10.1007/s10482-014-0376-0 25566956

[B268] Wang P. Kong F. Wei J. Wang Y. Wang W. Hong K.. (2014). Alkaloids from the Mangrove-derived actinomycete *Jishengella endophytica* 161111. Mar. Drugs. 12, 477–490. 10.3390/md12010477 24451190 PMC3917282

[B269] Wang X. Zhao J. Liu C. Wang J. Shen Y. Jia F.. (2013).*Nonomuraea solani* sp. nov., an actinomycete isolated from eggplant root *(Solanum melongena L.)*. Int. J. Syst. Evol. Microbiol. 63, 2418–2423. 10.1099/ijs.0.045617-0 23203622 PMC3749519

[B270] Weber T. Charusanti P. Musiol-Kroll E. M. Jiang X. Tong Y. Kim H. U.. (2015). Metabolic engineering of antibiotic factories: new tools for antibiotic production in actinomycetes. Trends Biotechnol. 33, 15–26. 10.1016/j.tibtech.2014.10.009 25497361

[B271] Wei Y. Z. Zhang Y. Q. Zhao L. L. Li Q. P. Su J. Liu H. Y. (2010). Isolation, screening and preliminary identification of endophytic actinobacteria from mangroves at Shankou of Guangxi Province. Microbiol. China. 37, 823–828.

[B272] Wu H. Chen W. Wang G. Dai S. Zhou D. Zhao H. (2012). Culture-dependent diversity of actinobacteria associated with seagrass (*Thalassia hemprichii*). Afr. J. Microbiol. Res. 6, 87–94. 10.5897/AJMR11.981

[B273] Xie Q. Y. Wang C. Wang R. Qu Z. Lin H. P. Goodfellow M.. (2011). *Jishengella endophytica* gen. nov., sp. nov., a new member of the family Micromonosporaceae. Int. J. Syst. Evol. Microbiol. 61, 1153–1159. 10.1099/ijs.0.025288-0 20543149

[B274] Xin Y. Huang J. Deng M. Zhang W. (2008). Culture-independent nested PCR method reveals high diversity of actinobacteria associated with the marine sponges *Hymeniacidon perleve* and *Sponge* sp. Antonie van Leeuwenhoek. 94, 533–542. 10.1007/s10482-008-9270-y 18670903

[B275] Xing K. Bian G. K. Qin S. Klenk H. P. Yuan B. Zhang Y. J.. (2012). *Kibdelosporangium phytohabitans* sp. nov., a novel endophytic actinomycete isolated from oil-seed plant *Jatropha curcas* L. containing 1-aminocyclopropane-1-carboxylic acid deaminase. Antonie van Leeuwenhoek 101, 433–441. 10.1007/s10482-011-9652-4 21989686

[B276] Xing K. Liu W. Zhang Y. J. Bian G. K. Zhang W. D. Tamura T.. (2013). *Amycolatopsis jiangsuensis* sp. nov., a novel endophytic actinomycete isolated from a coastal plant in Jiangsu, China. Antonie van Leeuwenhoek 103, 433–439. 10.1007/s10482-012-9823-y 23053697

[B277] Xing K. Qin S. Fei S. M. Lin Q. Bian G. K. Miao Q.. (2011). *Nocardia endophytica* sp. nov., an endophytic actinomycete isolated from the oil-seed plant Jatropha curcas L. Int. J. Syst. Evol. Microbiol. 61, 1854–1858. 10.1099/ijs.0.027391-0 20817835

[B278] Xing K. Qin S. Zhang W. D. Cao C. L. Ruan J. S. Huang Y.. (2014). *Glycomyces phytohabitans* sp. nov., a novel endophytic actinomycete isolated from the coastal halophyte in Jiangsu, East China. J. Antibiot. 67, 559–563. 10.1038/ja.2014.40 24736858

[B279] Xiong Z. J. Zhang J. L. Zhang D. F. Zhou Z. L. Liu M. J. Zhu W. Y.. (2013). *Rothia endophytica* sp. nov., an actinobacterium isolated from Dysophylla stellata (Lour.) Benth. Int. J. Syst. Evol. Microbiol. 63, 3964–3969. 10.1099/ijs.0.052522-0 23710050

[B280] Xu D. B. Ye W. W. Han Y. Deng Z. X. Hong K. (2014). Natural products from mangrove actinomycetes. Mar. Drugs. 12, 2590–2613. 10.3390/md12052590 24798926 PMC4052306

[B281] Xu L. H. Zhang H. Zhang L. P. Xue Q. H. Zhang L. X. Xiong Z. (2010). Microbial Resources Science. Beijing: Acedemic Press.

[B282] Yamamura H. Ashizawa H. Nakagawa Y. Hamada M. Ishida Y. Otoguro M.. (2011). *Actinomycetospora iriomotensis* sp. nov., a novel actinomycete isolated from a lichen sample. J. Antibiot. 64, 289–292. 10.1038/ja.2011.15 21386850

[B283] Yan X. Li Y. Wang N. Chen Y. Huang L. L. (2017). *Streptomyces ginkgonis* sp. nov., an endophyte from Ginkgo biloba. Antonie Van Leeuwenhoek 111, 891–896. 10.1007/s10482-017-0987-3 29177601

[B284] Yandigeri M. S. Meena K. K. Singh D. Malviya N. Singh D. P. Solanki M. K. (2012). Drought-tolerant endophytic actinobacteria promote growth of wheat (*Triticum aestivum*) under water stress conditions. Plant Growth Regul. 68, 411–420. 10.1007/s10725-012-9730-2

[B285] Yang G. L. Yang L. F. Jiang M. G. Wu J. F. Gan G. H. Tuo L. (2015). Isolation, identification and bioactivity of endophytic actinomycetes from mangrove plants in Beilun River. J. Agri. Biotech. 23, 894–904.

[B286] Yang H. Zhang Z. Yan R. Wang Y. Zhu D. (2014). Draft genome sequence of *Streptomyces* sp. strain PRh5, a novel endophytic actinomycete isolated from Dongxiang wild rice root. Genome Announc. 2:e00012–14. 10.1128/genomeA.00012-14 24744320 PMC3990736

[B287] Yokose K. Ogawa K. Sano T. Watanabe K. Maruyama H. B Suhara Y. (1983). New α-amylase inhibitor, Trestatins, I. Isolation, characterization and biological activities of Trestatins, A., B and C. J. Antibiot. 36, 1157–1165. 6605333 10.7164/antibiotics.36.1157

[B288] Yu H. Zhang L. Li L. Zheng C. Guo L. Li W.. (2010). Recent developments and future prospects of antimicrobial metabolites produced by endophytes. Microbiol. Res. 165, 437–449. 10.1016/j.micres.2009.11.009 20116229

[B289] Zhang J. Wang J. D. Liu C. X. Yuan J. H. Wang X. J. Xiang W. S. (2014a). A new prenylated indole derivative from endophytic actinobacteria *Streptomyces* sp. neau-D50. Nat. Prod. Res. 28, 431–437. 10.1080/14786419.2013.871546 24443904

[B290] Zhang X. Gao Z. Zhang M. Jing F. Du J. Zhang L. (2016). Analysis of endophytic actinobacteria species diversity in the stem of *Gynura cusimbua* by 16S rRNA gene clone library. Microbiol. 85, 379–385. 10.1134/S0026261716030176

[B291] Zhang Y. G. Wang H. F. Alkhalifah D. H. M. Xiao M. Zhou X. K. Liu Y. H.. (2018). *Glycomyces anabasis* sp. nov., a novel endophytic actinobacterium isolated from roots of Anabasis aphylla L. Int. J. Syst. Evol. Microbiol. 68, 1285–1290. 10.1099/ijsem.0.002668 29485399

[B292] Zhang Y. J. Zhang W. D. Qin S. Bian G. K. Xing K. Li Y. F.. (2013). *Saccharopolyspora dendranthemae* sp. nov. a halotolerant endophytic actinomycete isolated from a coastal salt marsh plant in Jiangsu, China. Antonie van Leeuwenhoek 103, 1369–1376. 10.1007/s10482-013-9917-1 23559043

[B293] Zhang Y. Liu H. Zhang X. Wang S. Liu C. Yu C.. (2014b). *Micromonospora violae* sp. nov., isolated from a root of Viola philippica Car. Antonie van Leeuwenhoek 106, 219–225. 10.1007/s10482-014-0184-6 24803239

[B294] Zhu W. Y. Zhang J. L. Qin Y. L. Xiong Z. J. Zhang D. F. Klenk H. P.. (2013). *Blastococcus endophyticus* sp. nov., an actinobacterium isolated from Camptotheca acuminata. Int. J. Syst. Evol. Microbiol. 63, 3269–3273. 10.1099/ijs.0.049239-0 23475341

[B295] Zhu W. Y. Zhao L. X. Zhao G. Z. Duan X. W. Qin S. Li J. (2012). *Plantactinospora endophytica* sp. nov., an actinomycete isolated from Camptotheca acuminate Decne., re-classification of Actinaurispora siamensis as Plantactinospora siamensis comb. nov. and emended descriptions of the genus Plantactinospora and Plantactinospora mayteni. Int. J. Syst. Evol. Microbiol. 62, 2435–2442. 10.1099/ijs.0.036459-0 22140153

[B296] Zin N. M. Baba M. S. Zainal-Abidin A. H. Latip J. Mazlan N. W. Edrada-Ebel R. (2017). Gancidin, W., a potential low-toxicity antimalarial agent isolated from an endophytic *Streptomyces* SUK10. Drug Des. Devel. Ther. 11, 351–363. 10.2147/DDDT.S121283 28223778 PMC5308589

